# Chronic DNA Replication Stress Reduces Replicative Lifespan of Cells by TRP53-Dependent, microRNA-Assisted MCM2-7 Downregulation

**DOI:** 10.1371/journal.pgen.1005787

**Published:** 2016-01-14

**Authors:** Gongshi Bai, Marcus B. Smolka, John C. Schimenti

**Affiliations:** 1 Department of Biomedical Sciences, Cornell University, Ithaca, New York, United States of America; 2 Department of Molecular Biology and Genetics, Cornell University, Ithaca, New York, United States of America; 3 Weill Institute for Cellular and Molecular Biology, Cornell University, Ithaca, New York, United States of America; 4 Center for Vertebrate Genomics, Cornell University, Ithaca, New York, United States of America; University of Washington School of Medicine, UNITED STATES

## Abstract

Circumstances that compromise efficient DNA replication, such as disruptions to replication fork progression, cause a state known as DNA replication stress (RS). Whereas normally proliferating cells experience low levels of RS, excessive RS from intrinsic or extrinsic sources can trigger cell cycle arrest and senescence. Here, we report that a key driver of RS-induced senescence is active downregulation of the Minichromosome Maintenance 2–7 (MCM2-7) factors that are essential for replication origin licensing and which constitute the replicative helicase core. Proliferating cells produce high levels of MCM2-7 that enable formation of dormant origins that can be activated in response to acute, experimentally-induced RS. However, little is known about how physiological RS levels impact MCM2-7 regulation. We found that chronic exposure of primary mouse embryonic fibroblasts (MEFs) to either genetically-encoded or environmentally-induced RS triggered gradual MCM2-7 repression, followed by inhibition of replication and senescence that could be accelerated by MCM hemizygosity. The MCM2-7 reduction in response to RS is TRP53-dependent, and involves a group of *Trp53*-dependent miRNAs, including the *miR-34* family, that repress MCM expression in replication-stressed cells before they undergo terminal cell cycle arrest. *miR-34* ablation partially rescued MCM2-7 downregulation and genomic instability in mice with endogenous RS. Together, these data demonstrate that active MCM2-7 repression is a physiologically important mechanism for RS-induced cell cycle arrest and genome maintenance on an organismal level.

## Author Summary

Duplication of the genome by DNA replication is essential for cell proliferation. DNA replication is initiated from many sites (“origins”) along chromosomes that are bound by replication licensing proteins, including MCM2-7. They are also core components of the replication helicase complex that unwinds double stranded DNA to expose single stranded DNA that is the template for DNA polymerase. Eukaryotic DNA replication machinery faces many challenges to duplicate the complex and massive genome. Circumstances that inhibit progression of the replication machinery cause “replication stress” (RS). Cells can counteract RS by utilizing “dormant” or “backup” origins. Abundant MCM2-7 expression sufficiently licenses dormant origins, but reducing MCMs compromises cellular responses to RS. We show that MCM2-7 expression is downregulated in cells experiencing chronic RS, and this depends on the TRP53 tumor suppressor and microRNAs it regulates. Extended RS eventually reduces MCMs to a point that terminal cell cycle arrest occurs. We propose that this mechanism is a crucial protection against neoplasia.

## Introduction

In preparation for DNA replication, “licensing” of replication origins occurs during late M to early G1 phase [1, 2]. These replication origins are selected and bound by the origin recognition complex (ORC) [3]. ORCs further recruit CDC6 and CDT1 to eventually load the MCM2-7 heterohexameric complex onto replication origins, thus forming pre-replication complexes (pre-RCs) [4]. Pre-RC formation is tightly regulated so origin licensing can only occur before, and not during, S phase to prevent re-replication of genomic regions [5]. Chromatin becomes replication-competent after MCM2-7 loading. Later, during S phase, replication machinery assembly is initiated at selected licensed origins with the formation of Cdc45/MCM2-7/GINS (CMG) replicative helicase complex, of which MCM2-7 is the catalytic core [6, 7]. Stable MCM2-7 chromatin association is required for uninterrupted replication fork progression and restart after stalling [8–10]. MCM2-7 is the sole complex present in both the pre-RCs and the active replisome, making it a nexus of DNA replication control.

The genome is vulnerable to exogenous and endogenous genotoxic stresses during DNA replication, which can lead to replication fork stalling [11]. Stalled replisomes must be stabilized to enable restart or displacement by converging replication forks to ensure complete and faithful DNA replication. Otherwise, mutations, genomic instability, and ultimately neoplasia can occur [12]. Numerous mechanisms exist to promote error-free replication under stressful conditions [13]. One of the mechanisms is utilization of dormant replication origins [11]. Most growing cells produce abundant amounts of MCM2-7 proteins that license large numbers of replication origins, but only a small proportion of these are used and they are sufficient to accomplish whole genome replication. This role of dormant origins in responding to RS was revealed in experiments where licensing was severely inhibited in cultured cancer cells via knockdown of MCM levels. While such cells can sustain limited proliferation under unchallenged conditions, the reduction of dormant origins renders them sensitive to additional RS [14–17]. Thus, abundant MCM production ensures adequate licensing of the dormant replication origins that serves as ‘backups’ and can be activated in response to stalled or collapsed replication forks and ensures completeness of DNA replication [18]. Inhibition of licensing in primary cells causes cell cycle arrest in G1 phase, leading to the proposed existence of a “licensing checkpoint” that prevents DNA replication under sub-optimal conditions [19, 20]. Thus, the physiological relevance of severe experimental conditions in transformed cell lines is unclear, and more importantly, little is known about endogenous MCM2-7 regulation in response to RS.

Another major mechanism that protects the genome during replication is the DNA damage response (DDR), components of which detect replication-associated lesions or cellular conditions that impair DNA replication. In addition to directly interacting with MCM2-7 subunits to stabilizing stalled replisomes, the DDR regulates cell cycle progression in response to RS, such as inducing senescence [21–23]. Central to this mechanism is the tumor suppressor gene *Trp53* (also called *Tp53* or *p53*). Genotoxic stress such as RS stabilizes TRP53, which then serves as transcriptional regulator of many downstream genes, including microRNAs (miRNAs) [24–26]. Through their complementary binding to one or more miRNA recognition elements (MREs) within the 3’ untranslated region (UTR) of a target protein-coding mRNA, miRNA-mRNA duplexes, together with the RNA-induced silencing complex (RISC), can negatively regulate gene expression, usually in a moderate manner [27, 28] that is sometimes reversible [29, 30]. Cells can exploit this mechanism to tune responses to genotoxic stresses, without committing to terminal cellular decisions such as apoptosis and senescence.

As mentioned earlier, most studies of how RS impacts cell growth and the DDR involve treatment of cell lines with exogenous agents that hinder DNA replication. One model of intrinsic RS is the *Mcm4*^*Chaos3*^ mutation in mice. This allele encodes a single amino acid change (Phe345Ile) that causes high levels of genomic instability and cancer susceptibility [31]. The "*Chaos3"* mutation destabilizes the MCM2-7 heterohexamer by disrupting MCM4-MCM6 interaction *in vitro* and is accompanied by a 40~60% decrease in MCM2-7 levels, leading to the conclusion that the associated phenotypes were primarily attributable to insufficient licensing of dormant replication origins [31–34]. This view is supported by similar cancer predisposition phenoptypes in mice that are hypomorphic for *Mcm2*, and which also show premature aging and stem cell defects in certain cell lineages [35, 36].

Here, we compare the consequence of low level endogenous RS in *Chaos3* cells to chemically-induced RS in WT cells. Our results indicate that RS induces TRP53-dependent MCM2-7 downregulation, which coincide with loss of DNA replication potential in primary cells that eventually becomes senescent. We also identified a group of *Trp53*-responsive miRNAs that regulates MCM expression in response to RS. Modulation of miRNA expression partially rescued RS-induced cellular defects, including MCM repression and genomic instability. Our observations reveal that active MCM2-7 regulation is a key aspect of senescence induction when cells are exposed to chronic low level RS, and is important for safeguarding organisms from cells that undergo potentially deleterious RS-induced genomic alterations.

## Results

### Cells with intrinsic RS senesce prematurely in culture

**Fig 1:**
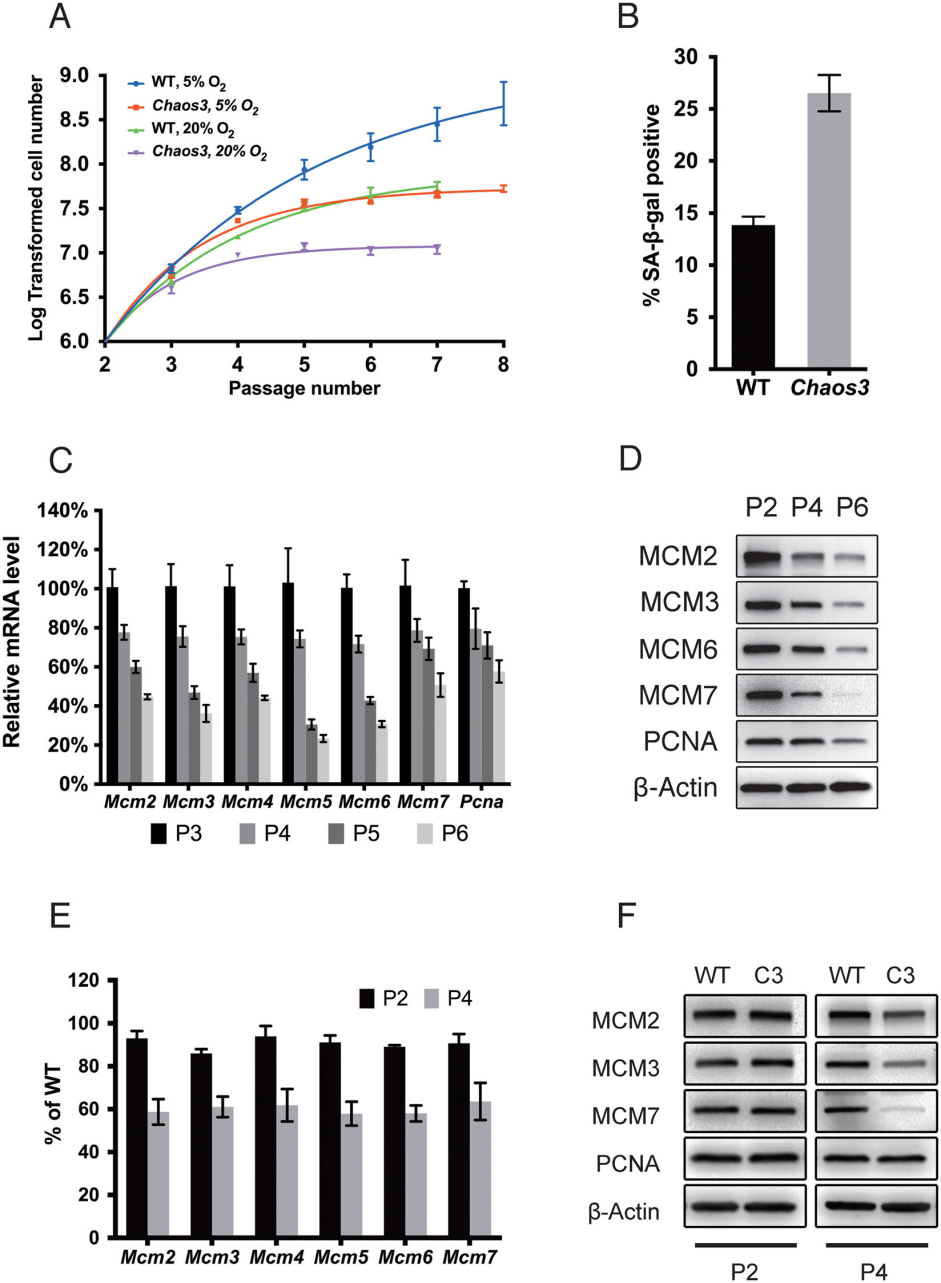
MCM2-7 pan-reduction accompanies senescence in primary MEFs, and both are accelerated by *Chaos3*. **(A)** Replicative lifespans of *Chaos3* and WT primary MEFs. Cells were maintained under the indicated O_2_ levels. Error bar = SEM. **(B)** Senescence is accelerated in *Chaos3* MEFs. The % of cells positive for SA-β-gal (senescence-associated β-galactosidase) was assessed at passage 5. Error bar = SEM. **(C)** *Mcm2-7* and *Pcna* mRNA levels in WT MEFs decline precipitously with extended culture. The level of each gene was measured by qRT-PCR. The mRNA level of each at P2 is considered to be 100%. At least 3 biological replicated were performed for each data point. Error bar = SEM. P = passage. **(D)** Western blot analysis of total cellular MCM protein during passage of primary WT MEFs. Immunoblots were probed with the indicated antibodies. **(E)** *Mcm2-7* mRNA and **(F)** protein levels decline more rapidly in primary *Chaos3* MEFs than WT MEFs during culture. Note that PCNA was unchanged between genotypes at both time points, while MCMs decreased between P2 and P4.

Early passage *Mcm4*^*Chaos3/Chaos3*^ ("*Chaos3*") MEFs have proliferation defects in the presence of aphidicolin, a DNA polymerase inhibitor [31]. To determine if this reflects a predisposition to senescence, we monitored the growth of freshly isolated wild-type (WT) and *Chaos3* MEFs for several passages, under typical culture conditions (20% O_2_). The *Chaos3* cultures exhibited reduced growth and eventual arrest at earlier passages than WT cultures (Fig 1A). Furthermore, approximately twice as many cells in *Chaos3* MEF cultures were positive for senescence-associated β-galactosidase (SA-β-gal) expression than in WT cultures (Fig 1B).

Oxygen sensitivity and DNA replication stress are the two major causes of natural senescence in cultured primary mouse cells [37]. Low oxygen conditions (5% O_2_) resulted in faster growth of both WT and *Chaos3* MEFs compared to standard conditions (20% O_2_; Fig 1A). Whereas WT MEFs continued proliferating in 5% O_2_, this condition only delayed the onset of senescence of *Chaos3* MEFs before they stopped growing at P6-P7 (Fig 1A). The growth defect of *Chaos3* MEFs under normal and low oxygen conditions is similar to that of MEFs defective in the non-homologous end joining (NHEJ) pathway of DNA double strand break (DSB) repair [37]. In aggregate, these observations suggest that intrinsic RS caused by the *Chaos3* mutation, not hypersensitivity to oxidative stress, triggers premature senescence.

### Endogenous RS induces MCM2-7 pan-reduction in association with senescence progression

*Chaos3* MEFs were reported to have 40–60% less MCM2-7 protein compared to WT cells [32, 34]. The difference is also reflected at the mRNA level and is largely specific to the MCMs, not other DNA replication and cell cycle related genes [33]. Our observations that *Chaos3* cells senesce prematurely in culture prompted us to investigate the cause and effect relationships between RS, MCM2-7 regulation, and senescence.

To determine if lower MCM2-7 in *Chaos3* MEFs is a constitutive feature of these cells or related to senescence, we tested whether WT primary MEF cultures exhibited MCM downregulation levels during passaging. The mRNA and protein levels of each MCM declined as a function of time in culture (Fig 1C and 1D). Levels of PCNA, another essential DNA replication protein, also declined albeit less dramatically than MCMs (Fig 1C and 1D, and S1 Fig). Decreased MCM levels have also been observed in older mouse hematopoietic stem cells (HSCs), which have increased RS [38].

We further measured MCM2-7 mRNA and protein in primary *Chaos3* and WT MEFs at both early (P2) and later (P4) passages. Consistent with the aforementioned published reports [32, 34, 39], we observed 40% less MCM2-7 in P4 *Chaos3* MEFs. However, there was little reduction of MCM2-7 mRNA or protein in P2 *Chaos3* MEFs compared to WT littermate MEFs (Fig 1E and 1F). These results suggest that MCM2-7 levels decrease roughly in parallel with the progression of RS-associated cellular senescence, and is either a cause or consequence of senescence. Furthermore, the results indicate that MCM2-7 pan-reduction in *Chaos3* cells is not an incipient property, but like in WT MEFs, is acquired and likely a consequence of RS from culture conditions, which is exacerbated or accelerated by the defective MCM4^Chaos3^ protein.

### The *Chaos3* mutation disrupts MCM association with replication forks and causes RS

**Fig 2:**
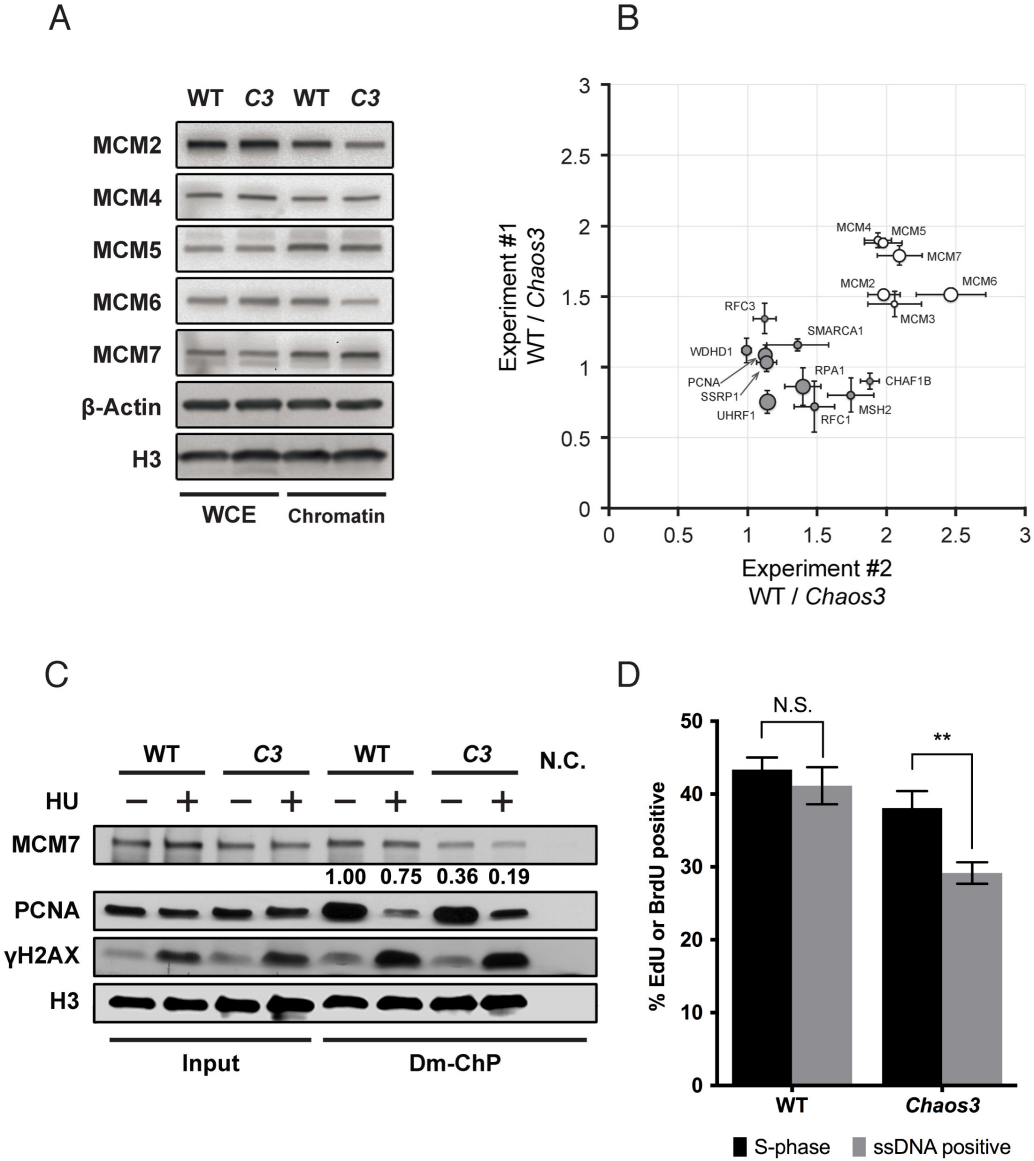
The *Chaos3* mutation disrupts MCM2-7 association with replication forks. **(A)** SV40 large T antigen immortalization of primary MEFs rescues MCM2-7 expression in *Chaos3* (C3) cells. Shown are western blots of whole cell proteins extract (WCE) and chromatin-bound (chromatin) fractions isolated from the WT and littermate *Chaos3* immortalized MEFs. The slightly lower level of chromatin-bound MCM2 & 6 in C3 cells likely reflects helicase destabilization or an excess amount of these subunits relative to the others. β-Actin and histone H3 serve as loading controls for total and chromatin-bound proteins, respectively. **(B)** Decreased MCM2-7 association at unchallenged replication forks in *Chaos3* mutant MEFs. SILAC labeling was performed in immortalized WT and *Chaos3* littermate MEFs followed by Dm-ChP and mass spectrometry. The data represents two experiments, each involving two different cell lines for each genotype. The isotope labeling was reversed in the two SILAC experiments. Relative protein quantity ratios (WT/*Chaos3* cells) were plotted for both experiments. The size of each circle represents the relative peptide enrichment overall in the MS analysis. Open circles are the six MCM2-7 proteins; filled circles are other indicated replication proteins. Error bar = SEM for each individual experiment. **(C)** The *Chaos3* mutation causes MCM2-7 disengagement from replication forks under RS. Shown are western blots containing proteins isolated before (“Input”) or after Dm-ChP. Both WT and *Chaos3* cells were SV40 T antigen-immortalized. Histone H3 signal in the Dm-ChP pull-down fraction represents amount of nascent DNA labeled during EdU incorporation, which serves as loading control. Accumulation of γH2AX and loss of PCNA from EdU-labeled nascent DNA after HU treatment reflects fork stalling and collapse, respectively [41]. Numbers below the MCM7 panel shows MCM7 retention relative to untreated WT cells. The numbers are generated after normalizing MCM7 to H3 loading control signal strength. N.C. = no ‘Click’ reaction performed during Dm-ChP. **(D)** *Chaos3* disrupts DNA replicative helicase function *in vivo* following polymerase arrest. This assay is designed to assess the fraction of cells bearing replication forks that have helicases capable of continued unwinding upon HU-induced stalling of the replicative polymerase. See S2 Fig for experimental design and examples of primary data. Immortalized WT and *Chaos3* cultures did not significantly differ in the fraction of actively replicating (S-phase) cells. The percentage of WT cells stained positively for BrdU under non-denaturing conditions (“ssDNA positive”) represent those with continued helicase unwinding after HU induced replication fork stalling. N.S. = not significantly different by two-sided t-test; ** represents a significant difference (p≈0.003).

To determine the primary source of RS in the *Chaos3* mutant, we investigated the biochemical nature of this mutation. The *Chaos3* Phe345Ile change disrupts MCM2-7 heterohexamer complex stability [33, 34] without reducing mutant helicase activity *in vitro* [34]. However, the *Chaos3* mutation causes spontaneous replication fork stalling *in vivo*, and some of these stalled forks go unprocessed and persist into M phase [34]. Since MCM2-7 complex integrity at replication forks is essential for replisome progression and stalled fork recovery/restart [8, 9], we hypothesized that the replication defect in the *Chaos3* cells is due to MCM2-7 helicase instability that compromises its association with replication forks. To test this, we studied MCM2-7 association at active and stalled replication sites using DNA-mediated chromatin pull-down (Dm-ChP) [40]. This technique isolates proteins bound to nascent DNA (labeled with 5-ethynyl-2-deoxyuridine, EdU). SV40 large T antigen-immortalized WT and *Chaos3* primary MEFs (isolated from littermates) were first subjected to stable isotope labeling of amino acid in culture (SILAC) to enable quantitative mass spectrometry (MS) analysis. Interestingly, total MCM2-7 levels in the SV40-immortalized *Chaos3* cells were not decreased as in primary *Chaos3* MEFs (Fig 2A). However, MCM2-7 association with nascent DNA was consistently reduced in the *Chaos3* samples (~50% reduction from two independent experiments; Fig 2B). Many other known replication proteins were also identified in this MS analysis, and their levels were similar in the WT and *Chaos3* samples. These observations indicate that the *Chaos3* mutation compromises MCM2-7 heterohexamer association with active replication forks *in vivo*.

Next, we assessed MCM2-7 retention at stalled replication forks by first labeling ongoing forks in immortalized MEFs with an EdU pulse, then adding a high concentration of the ribonucleotide reductase inhibitor hydroxyurea (HU) for 30 minutes to stall replication forks [41], followed by protein isolation using Dm-ChP. In both WT and *Chaos3* cells, HU caused an increase in γH2AX and decrease of PCNA, consistent with replication fork stalling [41]. About 25% of MCM7 dissociated from stalled replication forks in WT cells (Fig 2C; Dm-ChP “-” *vs*. “+” HU lanes), consistent with results using the similar iPOND method [42], compared to a 47% MCM7 loss in *Chaos3* cells (Fig 2C). This loss from stalled forks is in addition to ~3 fold decreased MCM7 association with unchallenged replication forks (Fig 2C; WT *vs*. *Chaos3* Dm-ChP lanes,—HU).

As expected, disengagement of MCM from stalled replication forks in the *Chaos3* cells disrupted helicase function, as visualized by immunofluorescence analysis of cells that were pulse-chased with BrdU. DNA polymerase stalling (by HU) leads to their uncoupling from the helicase, which normally continues to unwind genomic double-stranded DNA (dsDNA), thus exposing extensive amounts of single-stranded DNA (ssDNA) in front of the fork [43]. Since the anti-BrdU antibody only recognizes BrdU in ssDNA, immunolabeling of cells under non-denaturing conditions reveals the degree of helicase activity [44]. Unlike WT cells, a significant fraction of the replicating *Chaos3* cells failed to display detectable ssDNA accumulation after HU-induced polymerase stalling (Fig 2D and S2 Fig). Taken together, our observations suggest that the *Chaos3* mutation causes RS by disrupting MCM2-7 complex stability, thus compromising helicase association and function during normal and challenged DNA replication.

### Chronic RS induces MCM2-7 reduction coincides with loss of DNA replication potential and senescence, but precedes downregulation of cell cycle regulators

Given our data indicating that *Chaos3* helicase instability induces RS, and MCM2-7 downregulation accompanies senescence in *Chaos3* cells, we postulated that MCM2-7 pan-reduction is an authentic cellular response to RS in general (not unique to *Chaos3*), and that it contributes to the RS-induced senescence.

**Fig 3:**
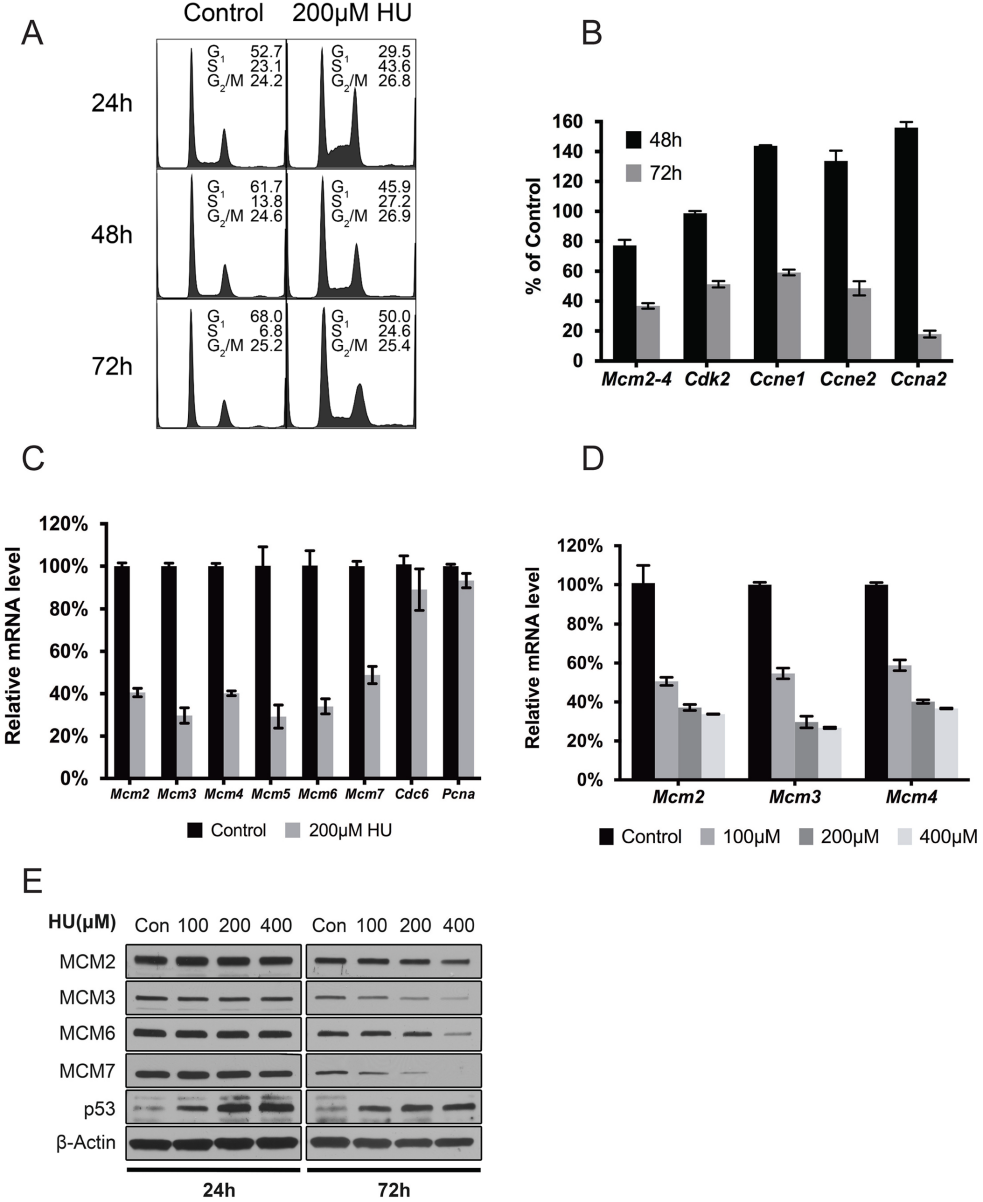
RS-induced MCM2-7 downregulation precedes loss of DNA replication. **(A)** Cell cycle profiles of WT primary MEFs under chronic RS. Plots are of DNA content as assessed by flow cytometry. The percentages of cells in each stage of the cell cycle are shown. Three technical repeats were performed. **(B)** qRT-PCR measurement of gene expression after 200μM HU treatment of WT primary MEFs for 48 or 72h. Plotted are mRNA levels after normalization to control samples receiving no HU treatment. **(C)** *Mcm2-7* specific repression after persistent RS treatment in early passaged WT primary MEFs. Plotted are mRNA levels as determined by qRT-PCR following 200μM HU treatment for 72h. Error bar = SEM. **(D)** Persistent RS induces MCM repression. *Mcm2-4* mRNA levels in WT primary MEFs were measured by qRT-PCR following 200μM HU treatment for the indicated periods of time. The values plotted are compared to untreated cells. Error bar = SEM. **(E)** Dosage-dependent *Mcm2-7* repression after persistent RS. *Mcm2-4* mRNA levels in WT primary MEFs were measured by qRT-PCR following 72h of culture in the indicated concentrations of HU. Error bar = SEM. **(F)** Induced RS reduces MCM protein expression in a time- and dose-dependent manner after HU treatment of WT primary MEFs. Shown are immunoblots of total protein extracted from primary MEFs exposed to the indicated concentrations of HU for 24 and 72h. Note TRP53 stabilization following HU treatment.

To test this, we treated early passage (P2) WT MEFs with a low concentration of HU as a means of imposing chronic RS. Long term HU treatment inhibited cell growth (S3A Fig) and caused an increase of cells in S phase (2–4 fold increase over untreated after 24-72h; Fig 3A). Despite this increase, DNA replication overall was inhibited due to HU treatment, as indicated by a relative lack (vs untreated) of EdU incorporation immediately after HU removal (S3B Fig). However, mRNA levels of genes governing S phase entry and progression were actually upregulated (cyclins *Ccne1*, *Ccne2* and *Ccna2*) or unchanged (cyclin dependent kinase 2, *Cdk2*) after 48h of HU treatment, while MCM mRNA levels had already declined by >20% (Fig 3B and S3C Fig). It wasn’t until more prolonged treatment (72h) that levels of the cyclins and *Cdk2* declined dramatically along with MCMs (Fig 3B), although expression of the essential replication gene *Pcna* and the licensing factor *Cdc6* were not affected (Fig 3C). This exclusivity of Mcm2-7 downregulation with respect to other replication/licensing genes mirrors that of untreated *Chaos3* MEFs [33]. The MCM2-7 pan-reduction was dependent upon duration and dosage of HU treatment. Longer exposure (S3C Fig) or increased concentration of HU (Fig 3D and 3E) caused greater MCM2-7 reduction. These results suggested that the degree of *Mcm2-7* downregulation is directly related to cellular responses to RS, which coincides with loss of DNA replication in primary cells.

**Fig 4:**
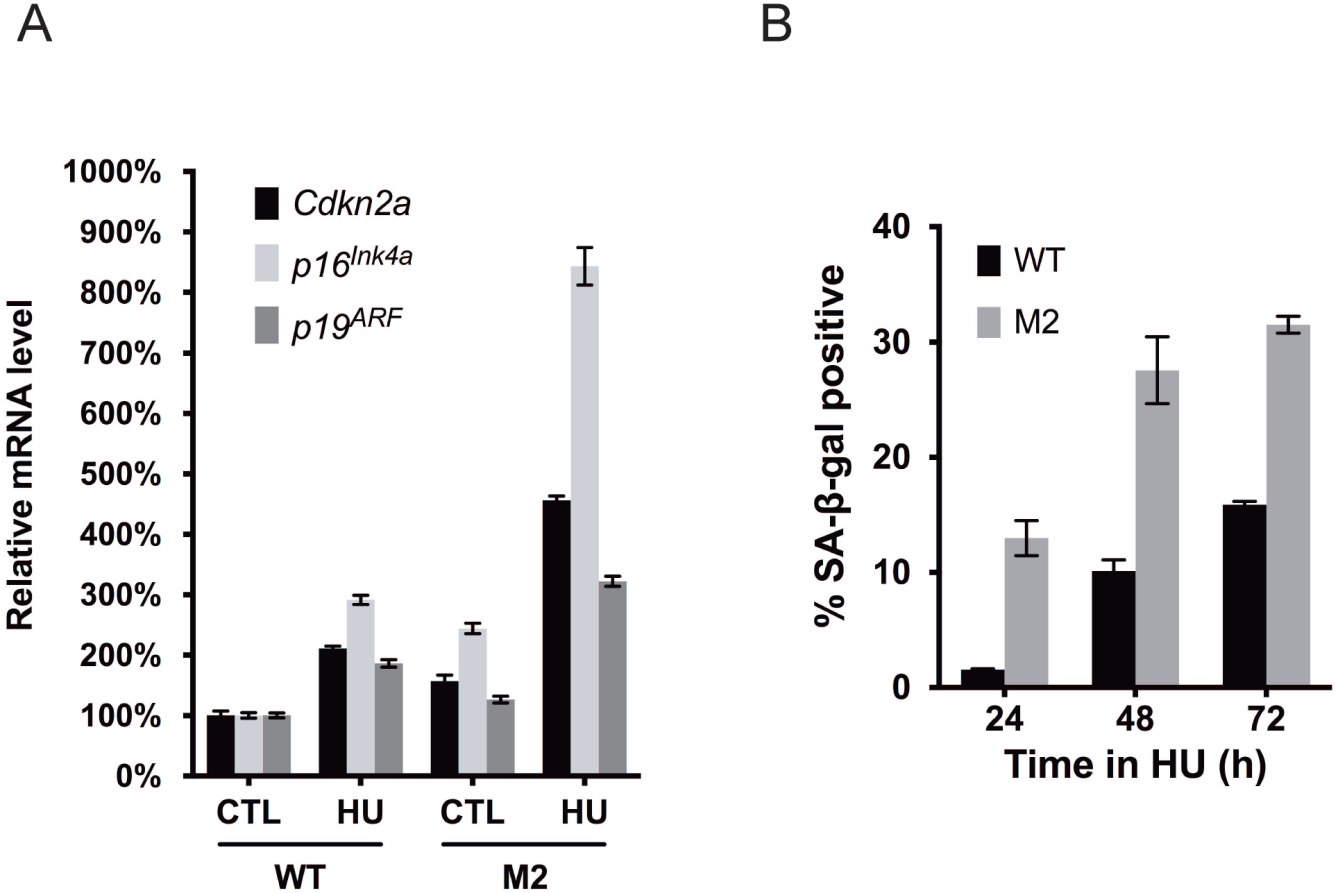
MCM haploinsufficiency sensitizes cells to RS-induced senescence. **(A)** Replication-stressed MEFs heterozygous for *Mcm2* exhibit dramatic upregulation of senescence-related transcripts. Cells of the indicated genotypes were exposed (or not) to 200μM HU for 72h. Primers specific to the *p16*^*Ink4a*^ and *p19*^*ARF*^ products of the *Cdkn2a* locus, as well as the primary transcript of *Cdkn2a* were quantified by qRT-PCR and normalized to β-actin levels. M2 = *Mcm2*^*Gt/+*^, where "Gt" indicates that this is a gene trap allele that is functionally null [32]. Error bar = SEM. **(B)** MEFs heterozygous for *Mcm2* are hypersensitive to RS-induced senescence. Cells were treated with 200μM HU for the indicated times. M2 = *Mcm2*^*Gt/+*^. The percentage of cells positive for SA-β-gal staining is shown for each sample. Error bar = SEM.

We next evaluated if HU treatment (72h) induced premature senescence of primary WT MEFs. This triggered a 2–3 fold increase of the *p16*^*Ink4a*^ and *p19*^*ARF*^ tumor suppressors (Fig 4A) which are associated with senescence and can be induced in response to persistent RS [45], and also a ~5–7 fold increase in the percentage SA-β-gal positive cells. The latter became evident after 48hrs of treatment (Fig 4B), concurrent with MCM reductions. These combined data indicate that chronic RS triggers a decrease in MCM levels and an increase in senescence markers before downregulating cell cycle regulatory genes or other DNA replication licensing and replication-related genes. However, these experiments did not reveal the order or causality of MCM downregulation vs senescence onset.

### Reduced MCM dosage sensitizes cells to RS and senescence in a TRP53-dependent manner

The results presented thus far show that MCM2-7 levels are reduced in response to intrinsic or chemically-induced RS, ultimately leading to reduced or abolished proliferation. Previous reports have indicated that severe reduction of an individual MCM sensitizes cancer cell lines to acute RS and impaired proliferation [15–17]. However, with respect to senescence, immortalized cells cannot address the relative contributions of exogenous RS *vs*. RS from physiologically-relevant MCM decreases. To test this, we examined proliferation and senescence of primary MEFs heterozygous for *Mcm2* ("M2") cultured with or without chemically-induced RS. M2 cells proliferated identically to WT littermate cultures under standard culture conditions, and did not senesce prematurely (S4 Fig). However, HU treatment triggered more severe senescence-associated phenotypes in M2 than WT primary MEFs, both in terms of markedly higher *p16*^*Ink4a*^*/p19*^*ARF*^ induction (Fig 4A) and a ~2 fold increase in cells positive for SA-β-gal (Fig 4B). Interestingly, M2 primary MEFs expressed slightly higher basal levels of senescence markers than WT MEFs (Fig 4A and 4B). Genetic reduction of MCM2 alone also causes a moderate MCM2-7 pan-reduction [32, 35, 36], supporting the idea that MCM reduction itself sensitizes cells to senescence.

**Fig 5:**
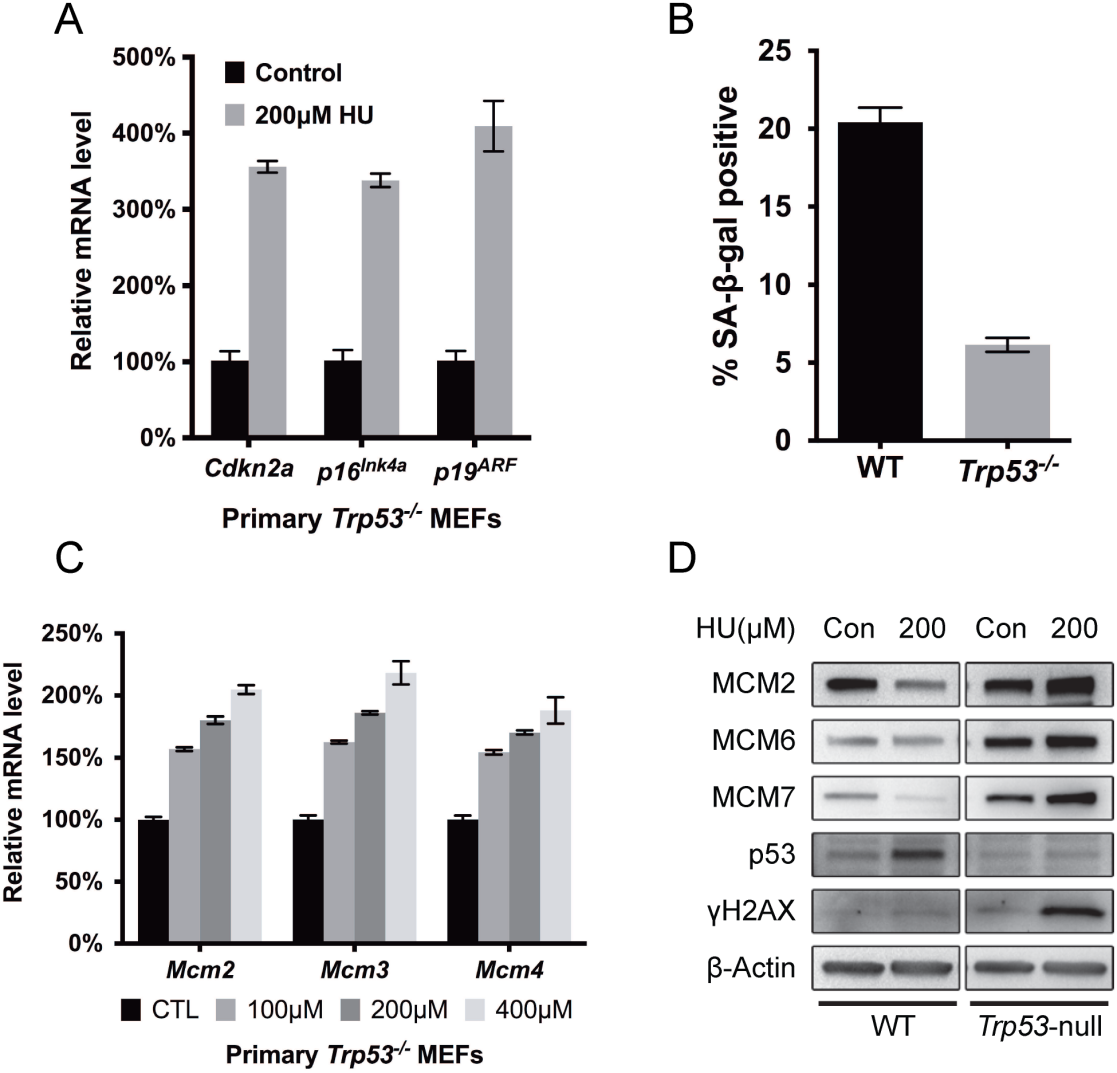
RS-induced MCM2-7 downregulation and cellular senescence is dependent upon TRP53. **(A)** RS treatment in primary *Trp53*^*-/-*^ MEFs upregulates senescence-associated transcripts similarly to WT MEFs. *Trp53*^*-/-*^ primary MEFS were treated (or not) with 200μM HU for 72h. Primers specific to the *p16*^*Ink4a*^ and *p19*^*ARF*^, products of the *Cdkn2a* locus, as well as the primary transcript of *Cdkn2a* were quantified by qRT-PCR and normalized to β-actin levels. **(B)** TRP53 deficiency prevents cellular senescence induction in response to chronic RS. Percentage of cells positive for SA-β-gal after 72 hours of exposure to 200μM HU is plotted. Error bar = SEM. **(C)** HU-induced RS causes *Mcm2-4* up-regulation in P2 *Trp53* null primary MEFs. Cells were cultured in the indicated concentrations of HU for 72h. Plotted are mRNA levels as assessed by qRT-PCR. Control cells received no HU, and these mRNA levels are considered to be 100%. Error bar = SEM. **(D)** RS induces DNA damage and increases MCM protein in *Trp53*-null primary MEFs. Cells were treated with 200μM HU for 72h, and then total protein was extracted for immunoblotting with antibodies against the indicated proteins. The phosphorylated form of H2AX (γH2AX) is an indicator of double strand breaks and replication fork stalling.

The DNA damage response (DDR) is essential for senescence induction in MEFs [37]. Since our data show that MCM2-7 repression is an authentic cellular response to RS and contributes to senescence induction, it is possible that MCM regulation is controlled by the DDR. Indeed, accumulation of TRP53 was observed in the unchallenged *Chaos3* cells, while *Trp53* deletion in this mutant rescues MCM2-7 levels in MEFs [39], consistent with our observation that SV40 T antigen, which inhibits *Trp53*, rescued overall MCM2-7 expression (Fig 2A) [46]. We postulated that if the intrinsic replication stress caused by the *Chaos3* mutation is what triggers TRP53-mediated MCM2-7 pan-reduction and premature senescence in *Chaos3* MEFs, then TRP53 would also mediate senescence induction and MCM2-7 repression in WT MEFs subjected to exogenous RS. Similar to WT primary MEFs (Fig 4A), HU treatment also stimulated *p19*^*ARF*^ expression in *Trp53*-null primary MEFs (Fig 5A). However, as ARF functions upstream of TRP53 to induce senescence in primary mouse cells [22], the terminal senescence phenotype is bypassed in *Trp53*-null MEFs, as indicated by the barely detectable SA-β-gal expression after RS induction (Fig 5B). Surprisingly, whereas HU treatment for 72 hrs caused a decrease in MCM mRNA in WT MEFs (Fig 3E), the identical treatment caused a dose-dependent 50–100% increase of *Mcm2*, *3* and *4* mRNA in *Trp53*-deficient cells treated with increasing concentrations of HU (Fig 5C). These increases were also reflected at the protein level (Fig 5D), unlike WT. Interestingly, HU-treated *Trp53*-null MEFs exhibited a drastic increase in H2AX Ser139 phosphorylation (γH2AX) compared to WT cells receiving the same treatment (Fig 5D), indicating greatly elevated DNA damage and/or replication fork errors, and likely reflecting a failure of cells that have accumulated such defects to undergo senescence/apoptosis. Together, these results suggest that TRP53 repression of MCM2-7 in damaged or stressed primary cells is important for an organism to minimize the persistence of cells with excessive genomic instability that could predispose to neoplasia.

### *Trp53*-dependent microRNAs represses MCM2-7 expression in response to RS

We previously reported that MCM2-7 repression in *Chaos3* cells occurs at the post-transcriptional level, is dependent upon *Drosha* and *Dicer*, and is paralleled by an increased levels of the *miR-34* family of microRNAs [33]. Those data, considered in conjunction with the results presented thus far, suggest that miRNA-related silencing plays a role in repressing MCM2-7 in response to RS.

**Fig 6:**
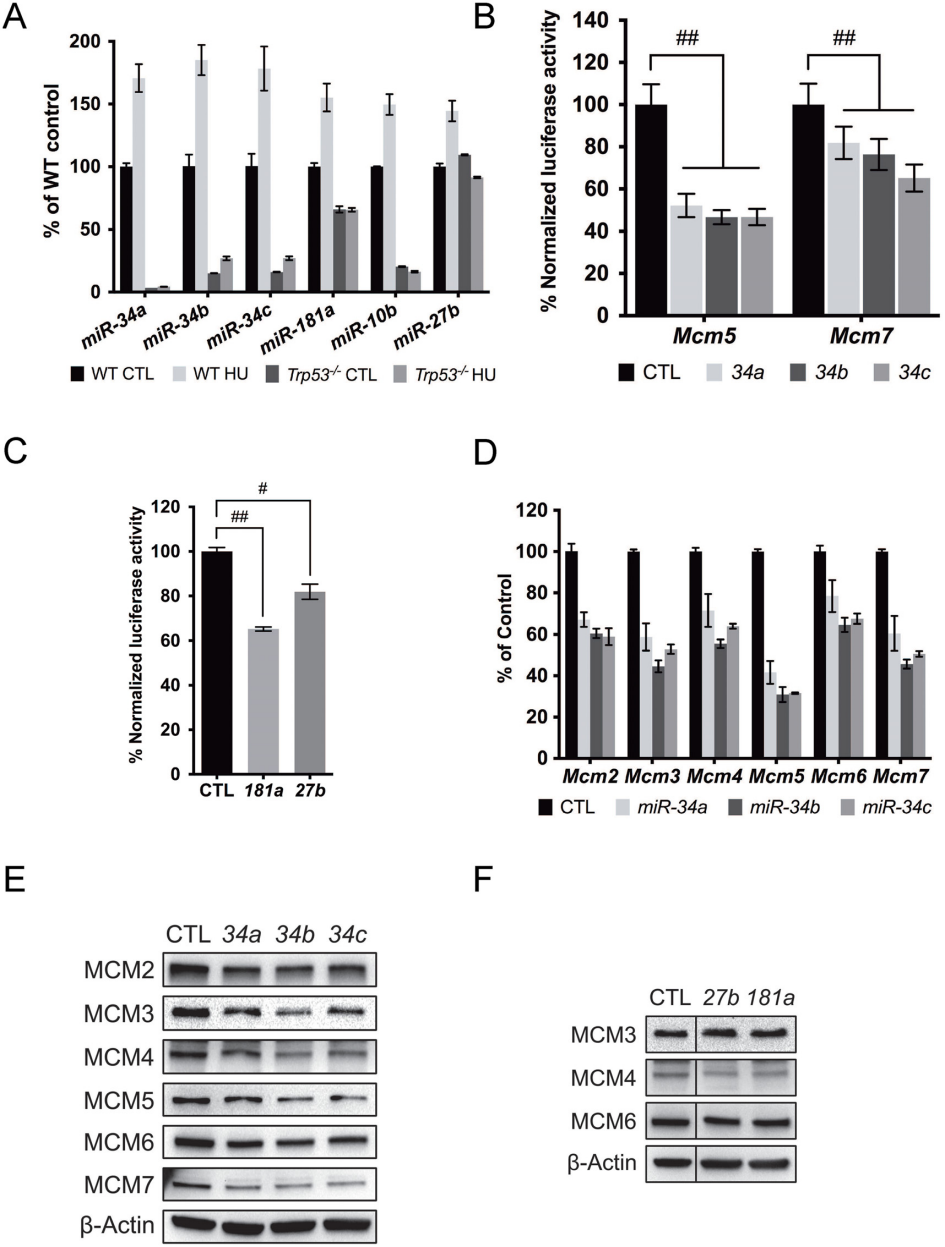
RS responsive miRNAs repress MCM2-7 expression. **(A)** RS induced miRNA upregulation depends on *Trp53*. miRNA levels were quantified by qRT-PCR and normalized to U6 small nuclear RNA (RNU6). The HU treatment was 200μM for 72h. Error bar = SEM. CTL = control. **(B)** *miR-34abc* acts upon the 3′UTR of *Mcm5* & *7* in HeLa cells co-transfected with a dual luciferase reporter bearing the full-length 3′UTR of the genes. *miR-34a*, *34b*, and *34c* were transfected individually and assays were performed after 48h. **(C)** Same as (B), but the impact of co-transfecting *miR-181a* or *miR-27b* with a reporter containing the full-length 3’UTR of *Mcm4* was assessed. **(D)** *miR-34abc* overexpression through miRNA mimic transfection individually caused reduced *Mcm2-7* mRNA expression. mRNA level of each gene was measured by qRT-PCR and normalized to β-actin levels. *Mcm2-7* mRNA levels were considered 100% in the control cells which were transfected with negative control miRNA mimics (based on cel-miR-67). Error bar = SEM. **(E)** Immunoblot showing MCM protein levels declined 48 hrs after *miR-34a*,*b* or *c* transfection. **(F)** Same as (E), but different miRNAs were transfected. Note that the control lane in (F) is the same as in (E).

To identify the culprit miRNAs, we performed small RNA sequencing on RNA samples isolated from WT primary MEFs after HU treatment, and on RNA samples isolated from WT and *Chaos3* primary MEFs at each passage (P2~P5). Among the miRNAs that are significantly upregulated by endogenous and exogenous RS, we found *miR-10b*, *27b*, *181a* and all the members of the *miR-34* family miRNAs. We confirmed the sequencing results using miRNA qRT-PCR. Interestingly, *Trp53* deletion abolished the HU induced miRNA upregulation in the WT primary MEFs (Fig 6A), confirming these candidate miRNAs are *Trp53*-dependent [25].

miRNAs usually interact with miRNA recognition elements (MREs) in the 3’UTR of the target mRNAs to repress their expression. Full-length 3’UTRs of each *Mcm2-7* gene were cloned into a dual-luciferase reporter plasmid. Overexpression of all the aforementioned miRNAs except *mir-10b* repressed luciferase activity regulated by the corresponding MCM 3’UTR, confirming *in silico* predictions (Fig 6B and 6C; see Methods). We also found potential targeting of the *Mcm7* 3’UTR by *miR-34*, despite the lack of *in silico*-predicted binding sites (Fig 6B). These results indicate that the candidate miRNAs target MCMs transcripts to reduce their expression, consistent with a subset of observations in human cells [47]. We also evaluated *Mcm2-7* mRNA and protein levels after miRNA over-expression. *Mcm2-7* mRNA levels were not reduced after *miR-10b*, *181a* or *27b* over-expression (S5B Fig), while *miR-27b* and *181a* repressed MCM4 but not other MCMs studied (Fig 6F). Although *miR-34* mainly repressed MCM5 as indicated by the luciferase assay (Fig 6B), supporting the finding that it is a direct target of *miR-34a* in the context of the RISC [47], overexpression of the *miR-34s* individually diminished MCM2-7 mRNA and protein (Fig 6D and 6E). Interestingly, siRNA knockdown of MCM5 also caused MCM2-7 pan-reduction (S5C Fig). In sum, we identified a group of *Trp53*-dependent miRNAs that can regulate MCM2-7 expression directly or indirectly in response to RS.

### *miR-34abc* knockout in *Chaos3* mice increases MCM2-7 expression and reduces genomic instability, but does not increase tumor-free survival

**Fig 7:**
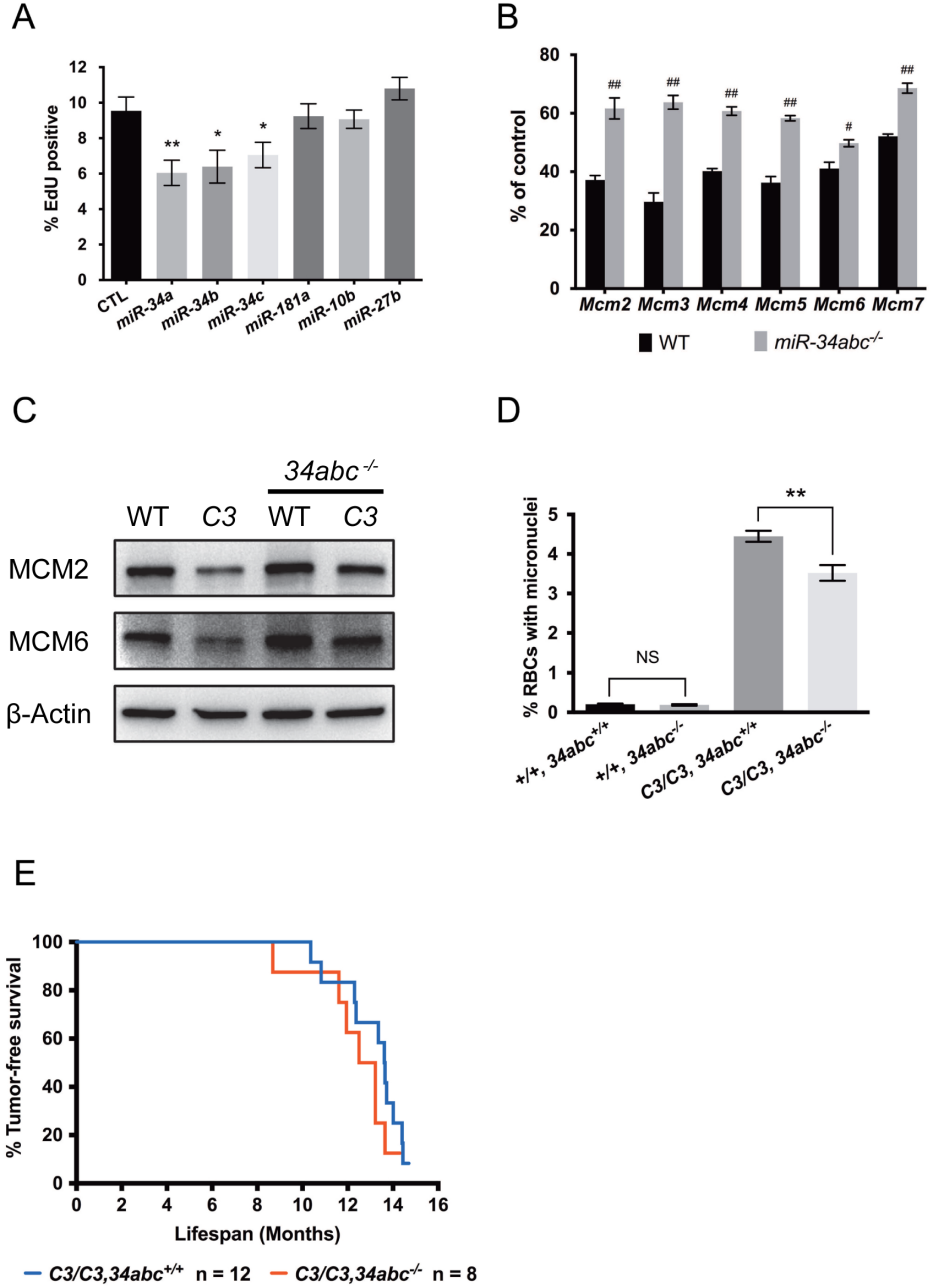
*miR-34abc* deficiency partially rescues RS-induced MCM2-7 repression and genomic instability in the *Chaos3* mutant. **(A)** Ectopic *miR-34* expression inhibits DNA replication. Primary WT MEFs transfected with *miR-34* miRNA mimics for 48h significantly inhibit DNA replication (two sided t-test, *, p<0.05, **, p<0.005), but not *miR-10b*, *27b* or *181a* mimics. Control cells were transfected with the negative control miRNA mimics. Error bar = SEM. **(B)** *miR-34abc* deletion partially rescues *Mcm2-7* mRNA levels after RS induction. WT and *miR-34abc*^*-/-*^ primary MEFs were treated with 200μM HU for 72h and *Mcm2-7* mRNA levels were measured by qRT-PCR. β-actin levels were also measured to normalize the *Mcm2-7* levels (statistically significant, two-sided t-test. #, p<0.01, ##, p<0.001). Error bar = SEM. **(C)** *miR-34abc* deficiency partially rescues MCM protein expression in liver from *Chaos3* (*C3/C3*), *miR-34abc*^*-/-*^ compound mutants. Total protein extracted from age-matched WT and mutants was immunoblotted to measure MCM expression. Protein expression of β-actin was used as loading control. **(D)** *miR-34abc* deletion in the *Chaos3* (“C3”) mutant significantly rescued micronuclei formation (two-sided t-test, **, p<0.005). Numbers of individuals analyzed for each genotype (from left to right) were 12, 5, 31 and 17 respectively. **(E)** Kaplan-Meier tumor-free survival plot of female mice with the indicated genotypes. Additional *miR-34abc* deletion did not affect tumor-free survival of the *Chaos3* (“C3”) mutant (Log-rank / Mantel-Cox test, p = 0.2600; Gehan-Breslow-Wilcoxon test p = 0.2356). Median tumor-free survival latency *C3/C3*, *34abc*^*+/+*^ = 13.63, *C3/C3*, *34abc*^*-/-*^ = 12.86 months.

Since MCM dosage impacts RS and *Trp53*-dependent RS-responsive miRNAs can regulate MCM expression, we tested whether modulating miRNA expression affects cellular responses to RS. Among the RS responsive miRNAs we studied, only the *miR-34* family miRNAs caused MCM2-7 pan-reduction upon ectopic expression (Fig 6D and 6E), a scenario similar to MCM2-7 repression after RS induction. Furthermore, overexpression of the *miR-34* miRNAs, but not other miRNAs, significantly inhibited DNA replication (Fig 7A). Numerous reports demonstrated that *miR-34* miRNAs impact cell cycle progression partly by targeting DNA replication genes, including MCMs [25, 47, 48]. To determine if these miRNAs impact cellular response to RS *in vivo*, we generated *miR-34abc* triple knockout (34TKO) mice, with and without the *Chaos3* mutation, and examined genomic instability and MCM levels in these mice and cells derived from them.

*miR-34* deletion partially rescued MCM2-7 pan-reduction in HU-treated primary WT MEFs (Fig 7B), complementing the previous experiments in which overexpression of *miR-34s* decreased MCM levels. To determine if *miR-34abc* deficiency could also rescue RS phenotypes *in vivo*, we measured MCM protein levels in various tissues. *Chaos3* mice had dramatically lower MCMs in multiple tissues compared to WT, similar to primary MEFs at later passages (Fig 7C; liver is shown). This observation indicates that the MCM2-7 pan-reduction in MEFs is not a culture artifact. MCM expression in both WT and *Chaos3* mice was increased by 34TKO (Fig 7C). These results indicate that *miR-34* expression contributes to both endogenous and exogenous RS-induced MCM2-7 repression *in vivo* and *in vitro*.

A hallmark of the *Chaos3* mutation is highly elevated micronuclei (MN), an indicator of genomic instability (GIN), in reticulocytes and erythrocytes [31]. *The* 34TKO reduced MN levels in *Chaos3* mice by ~20% (Fig 7D), and this reduction in MN was sensitive to *miR-34* genetic dosage (S6 Fig). However, *Chaos3 34TKO* females still succumbed to cancers with a latency similar to *Chaos3* single mutants (Fig 7E). These data, in conjunction with the results showing that *miR-34abc* ablation increased MCM levels in *Chaos3* mouse tissues, indicates that at least part of the genomic instability in *Chaos3* mice is related to decreased replication origin licensing orchestrated by TRP53 induction of MCM-targeting miRNAs. However, MCM reduction in the *Chaos3* mutant has a very minor impact on genomic instability and tumorigenesis, supporting our hypothesis that another character of the *Chaos3* mutation, probably the helicase instability, is what stimulates secondary mutations (namely deletions) that drive tumors in *Chaos3* mice [49].

## Discussion

In this report, we studied cellular responses to both intrinsic (*Chaos3* mutation) and environmentally-induced (HU treatment) RS in primary cultured mouse cells. Whereas normal cells maintain high MCM2-7 levels to sufficiently license dormant origins to cope with short-term RS, we found that chronic RS gradually decreases MCM2-7 expression over multiple generations in a *Trp53*-dependent manner. A group of *Trp53*-responsive miRNAs was identified which target MCMs directly to repress their expression in the presence of RS. Eventually, MCM levels become drastically lower, and proliferation ceases as senescence occurs. We postulate this is a consequence of failure to satisfy the hypothesized "licensing checkpoint," which requires a minimal level of licensed replication origins to enter S phase [20].

*Mcm2-7* are essential for DNA replication and their expression is highly correlated with the proliferation status of cells. High MCM expression is observed in proliferative cells, including tumor cells, but not in quiescent or terminally differentiated cells [50–52]. Severe knockdown of individual MCMs impairs proliferation of cultured cancer cells, indicating that the high levels are required for rapid proliferation [15–17]. Interestingly, hematopoietic stem cells (HSCs) isolated from old mice exhibit heightened RS attributable to selective down-regulation of MCM2-7 expression, and MCM knockdown in young HSCs impairs their expansion when transplanted into mice [38]. Genetically reducing *Mcm2* expression to about 1/3 of normal levels causes stem cell defects and cancer predisposition in mice [35, 36]. Furthermore, studies of mice in which MCM levels were reduced by combinatorial *Mcm* mutations led to the proposal of an MCM threshold, below which cell proliferation was compromised to a degree that developmental syndromes or embryonic lethality would occur [32]. Hypomorphic *MCM4* mutations causing reduced levels of WT transcripts have been discovered in humans and cause various developmental defects and genomic instability [53, 54]. Conversely, high level MCM expression is predictive of the oncogenic potential of pre-cancerous tissue, and is also used as an immunocytological marker to determine tumor grade and prognosis [19, 50, 51].

The prevailing model for why proliferating cells contain levels of MCM2-7 that exceed the amount required to license all primary origins and replicate the genome (also known as "MCM paradox") is that cells license dormant origins that can be activated to replicate sequences in which an adjacent replication fork has stalled or collapsed [55]. This model arose from studies in which severe MCM reduction was induced (for example by siRNA) in conjunction with intense environmentally-induced RS, demonstrating that dormant origins are especially important for maintaining genomic integrity under conditions of RS [15–17]. However, since these studies were performed on transformed cell lines lacking a normal DDR and cell cycle control, and involved extreme degrees of MCM and licensing perturbation, the relevance to normal physiological situations is unclear. Consistent with previous reports that also used primary cell cultures, we found that moderate MCM reduction neither affects unchallenged cell proliferation, nor triggers a significant replication-related DDR [34, 35]. However, our data showed that MCM reduction sensitizes the cells to additional RS as did Kunnev et al [35], and in primary cells, senescence is the terminal consequence of RS-induced cell cycle arrest. Importantly, we showed that moderate MCM reduction induces expression of senescence markers at a low level that is compatible with continued cell growth [37], but poises the cells to undergo full-scale senescence upon additional RS.

Our findings underscore the importance of the DDR and *Trp53* in response to RS, especially through their interaction with MCM2-7. Activated components of the DDR interact with MCM proteins to stabilize stalled replication forks and regulate dormant origin firing during periods of short-term RS [10, 56–58]. DDR-mediated activation of *Trp53* to arrest cell cycle progression and trigger senescence has been well studied [59]. TRP53 is also required for delaying premature S-phase entry when replication licensing is inhibited in normal primary cells [60]. However, this licensing checkpoint delay is bypassed in cancer cell lines that lack a normal DDR and/or *Trp53* function, leading to DNA damage accumulation from unregulated cell cycle progression and apoptosis [20, 60]. TRP53 function during the licensing checkpoint is thus indicated, and our findings support the role of TRP53-dependent regulation of licensing factors under RS. The regulatory function of TRP53 on MCM2-7 expression was first implicated by the observation that *Trp53* deletion rescued MCM2-7 levels in *Chaos3* MEFs [39]. We found that TRP53 represses MCM2-7 expression in cells subjected to HU-induced RS. *Trp53*-null cells continued proliferating under RS, presumably because senescence cannot be sufficiently induced.

We also identified a group of miRNAs that can be induced by RS, which also depends on normal *Trp53* function. Ectopic expression of the identified miRNAs suppressed MCM expression direct or indirectly. miRNA-related expression regulation is typically moderate [27, 28], and sometimes reversible [29, 30]. We found that primary MEFs exposed to low level RS took several generations to develop terminal phenotypes such as senescence. During this time, MCMs became gradually downregulated, consistent with the pattern of miRNA-related gene silencing. The *miR-34* family targets MCM5 directly, and overexpression of these miRNAs causes downregulation of other MCMs and DNA replication genes to negatively regulation cell cycle progression [25, 33, 47, 48]. Consistent with other studies showing that *miR-34s* function redundantly in the *Trp53* pathway [61, 62], we observed no apparent genomic instability defects in *miR-34*abc TKO mutants. Furthermore, *miR-34abc* deletion only partially rescued MCM expression, which in turn led to a partial decrease in genomic instability in *Chaos3* mice. These observations may have the following implications. First, reduced MCM2-7 expression in the *Chaos3* mutant may only contribute in a minor way to the highly elevated level of genomic instability in these cells/mice, whereas helicase instability is the major culprit. Second, other miRNAs may also repress MCM expression, as we showed was the case for *miR-27b* and *181a*. It is very likely that other mechanisms contribute to MCM regulation during RS.

These observations lead us to propose a model by which RS-triggered *Trp53* activation/stabilization lowers replication licensing factors to eventually arrest cell proliferation. Though moderate MCM reduction is tolerated in normal cells, chronic exposure to low-level RS can decrease MCM2-7 levels, and the degree of this downregulation is related to duration and intensity of the RS. An initial MCM reduction may not affect DNA replication and cell cycle progression, however, prolonged RS will continue to decrease MCM2-7 expression, partially through increased miRNA expression. Once MCMs decline below a threshold needed for licensing sufficient replication origins, the licensing checkpoint is implemented and cell proliferation is terminally arrested through senescence induction. This model provides an additional rationale for the high MCM2-7 expression in normal cells: that it is important for RS tolerance. This may have physiological relevance for stem cell pools. As mentioned before, severe MCM2 loss can lead to stem cell deficiency and arrested growth in mice [36]. The gradual loss of MCM2-7 expression in proliferative stem cells due to enduring prolonged and/or intense RS may dictate their replicative lifespans, thus these cells can be eliminated and the entire mechanism serving as a barrier to malignant transformation. To our knowledge, this is the first report to demonstrate active DNA replication control in terms of MCM regulation in response to RS in mammalian cells.

The *Chaos3* mutant is an unique model of intrinsic RS. Because MEFs homozygous for *Chaos3* exhibited ~40% MCM2-7 pan-reduction and reduced numbers of dormant origins, and since studies in other systems showed that reducing dormant origins causes genomic instability that is also characteristic of *Chaos3* mice and cells, we and others attributed the phenotypes to the shortage of dormant origins [31, 32, 34, 39]. Nevertheless, the trigger for MCM reduction is likely MCM2-7 heterohexamer destabilization; the F345I change in MCM4^Chaos3^ disrupts MCM4-MCM6 interaction *in vitro* and *in vivo* [33, 34]. Although this mutation does not affect helicase activity on naked DNA *in vitro* [34], it is likely to impact unwinding of certain chromatin structures *in vivo*; indeed, yeast bearing the *Chaos3* mutation showed chromosome breakpoints and rearrangements that were exclusively associated with Ty elements or solo long terminal repeat (LTR) elements [63]. Thus, we hypothesized that the genomic instability in *Chaos3* mice and cells might be due to helicase instability primarily, and that RS caused by the defective helicase triggered MCM2-7 pan-reduction, which in turn likely adds to genomic instability and increases RS.

This hypothesis was supported by the results of Dm-ChP experiments, a method for detecting protein dynamics at active and/or stalled replication forks [40, 41, 64]. These experiments revealed that *Chaos3* cells normally have no significant alterations in the levels of proteins associated with stalled replication forks, consistent with observations that markers of fork stalling and DNA damage (RPA32, pRAD17, γH2AX) are only moderately increased in *Chaos3* cells during unchallenged S-phase [34]. However, the accumulation of such proteins can still be induced upon RS. We also observed additional loss of MCMs at stalled replication forks in *Chaos3* cells. Normally, MCMs are retained at stalled forks to enable restart after repair [8, 10]. Given that additional MCM chromatin loading is prohibited once S-phase is initiated, the loss of MCM protein from stalled forks likely explains the unresolved replication intermediates interconnecting sister chromatids that persist into M phase in *Chaos3* cells [34].

In sum, we have found that RS-mediated downregulation of MCM2-7 levels, which occurs over a period of time that we surmise is related to providing cells with an opportunity to overcome RS, is a key mechanism for eventually inducing senescence in WT cells. This is likely an important way to prevent transformation of cells experiencing a certain threshold of genomic instability. However, a conundrum is why *Chaos3* mice or mice deficient for *Mcm2* [36] are cancer prone [31, 36], since they would be expected to be more susceptible to undergoing senescence. We conjecture that since there is a threshold level for MCMs below which causes lethality and severe developmental defects [32], that these mice are above that level and the cells that become transformed have acquired resistance to the senescence pathway, possibly *via* mutation or epigenetic alterations caused by intrinsic RS.

## Materials and Methods

### Ethics statement

The use of animals in this study was performed under a protocol (2004–0038) approved by Cornell’s IACUC. Mice were euthanized via CO_2_ asphyxiation according to IACUC-approved conditions.

### MEF preparation and proliferation assay

Primary MEFs were isolated from 12.5~14.5 dpc strain C3HeB/FeJ (C3H) embryos in which organs were removed and the remainder was lightly homogenized to make a cell suspension. Cells were cultured in DMEM with 10% FBS and 100 units/ml penicillin-streptomycin. The initial plating of embryonic cells is designated passage 0 (P0). For cell proliferation assays and general MEF maintenance, 1 x 10^6^ cells were seeded in 100 mm tissue culture dishes and maintained under either standard conditions (37°C, 5% CO_2_ and atmospheric O_2_) or low oxygen culture (37°C, 5% CO_2_ and 5% O_2_ level) in parallel for 3~4 days between passages. Upon trypsinization for passage, cell numbers were counted.

### Senescence-associated β-galactosidase (SA-β-gal) activity staining

SA-β-gal staining of cultured cells was performed as described [65]. To facilitate the counting of SA-β-gal positive cells, nuclei were counterstained with Hoechst 33342 before mounting the coverslips. The slides were examined using light and fluorescent microscopy.

### Quantitative RT-PCR (qRT-PCR)

Total RNA was extracted from cultured cells using the EZNA total RNA kit (Omega). cDNA was synthesized from 1ug of total RNA using the iScript cDNA synthesis kit (Bio-Rad) and the supplied oligo-dT primer. qPCR reactions were performed as described [32]. PCR amplification and real-time detection was performed with a Bio-Rad CFX96 Real-Time system and data analysis was performed with the Bio-Rad CFX Manager software (Bio-Rad). Relative gene expression was calculated using the ΔΔCq method with β-actin as endogenous control. A technical replicate was performed on each sample.

### microRNA quantitative RT-PCR (qRT-PCR)

Total RNA was extracted from cultured cells using miRNAeasy kit (Qiagen). cDNA of small RNA was synthesized from 1ug of total RNA using qScript microRNA cDNA Synthesis Kit (Quanta). PCR amplification and real-time detection was performed with a Bio-Rad CFX96 Real-Time system and data analysis was performed with the Bio-Rad CFX Manager software (Bio-Rad). Relative gene expression was calculated using the ddCq method with RNU6 as endogenous control. A technical replicate was performed on each sample. The primers for microRNA amplification were purchased from PerfeCTa microRNA assays (Quanta).

### Western blot analysis

Protein samples concentrations were determined with a BCA Protein Assay Kit (Thermo Scientific). Equal amounts of protein were loaded onto SDS-PAGE gels. Western blot analysis was performed as previously described [32, 33]. Proteins were electrotransferred onto PVDF membranes (Millipore). Chemiluminescence was performed using the Luminata HRP substrate (Millipore). Bands were detected either by exposure of the probed membranes to X-ray film or by scanning with a Bio-Rad Universal Hood II running Image Lab software (Bio-Rad). Western blot quantification was performed using ImageJ software.

Antibodies used were as follows: MCM2: ab108935 (Abcam); MCM3: 4012 (Cell Signaling); MCM6: sc-9843 (Santa Cruz Biotechnology); MCM7: ab2360 (Abcam); MCM7: 3735 (Cell Signaling); PCNA: P8825 (Sigma); total p53: 9282 (Cell Signaling); and β-actin: A1978 (Sigma).

### Flow cytometry and cell cycle profiling

Cells were trypsinized into a single cell suspension and fixed in 70% ice-cold ethanol overnight. They were stained for DNA content with 40μg/mL propidium iodide and 20μg/mL RNaseA for 30min at room temperature. Flow cytometry was performed on a BD Bioscience LSR II instrument. Stained cells were excited with a 488nm laser, and a 575/26 filter was applied for data collection. The percentages of cells in each cell cycle compartment was determined using ModFit LT software (Verity Software House).

### EdU incorporation assay

Cells grown on coverslips were pulse labeled with 10μM EdU for 30min. Formaldehyde was added directly to the culture to a final concentration of 1% for 10min at room temperature (RT). After 3 washes with PBS, cells were permeablized on ice with 0.3% Triton X-100 in PBS for 15 min., followed by 3 washes in PBS containing 1% BSA. The ‘Click’ reaction staining was performed by placing the cells in 10mM (+)-sodium-L-ascorbate, 0.1mM 6-Caboxyfluorescein-TEG azide and 2mM CuSO_4_ cocktail for 30 min at RT. After PBS washes, nuclei were counterstained with Hoechst 33342. Coverslips were mounted using ProLong Gold antifade reagent (Invitrogen) before examination by fluorescence microscopy.

### Protein extraction and cell fractionation

Whole cell protein extraction was performed in RIPA buffer. Protein extraction from tissues was performed using T-PER Tissue Protein Extraction Reagent (Thermo Scientific). For fractionation, cultured cells were trypsinized and counted. After two PBS washes, cells were resuspended in Buffer A (10mM HEPES [pH7.9], 10mM KCl, 1.5mM MgCl_2_, 340mM sucrose, 10% glycerol, 1mM DTT) with 0.1% Triton X-100 and incubated on ice for 5min. Low speed centrifugation (1,300g x 4min at 4°C) was performed to separate the supernatant (S1) and the pellet (P1). The pellet was washed once with Buffer A, then further extracted in Buffer B (3mM EDTA, 0.2mM EGTA, 1mM DTT) on ice for 30min. After centrifugation (1,700g x 4min at 4°C), the supernatant contained the nuclear fraction (S3), and the pellet (P3) containing the chromatin-bound proteins was washed once with Buffer B and then finally extracted in RIPA buffer. During the fractionations, cells were resuspended at 2.5 x 10^4^ /μL at each step.

### DNA mediated chromatin pull-down (Dm-ChP)

Dm-ChP was performed essentially as described [40] with the following modifications. EdU pulse labeling was performed for 30 min. for all experiments. One mg of nuclear-enriched protein lysate was incubated with 100μL pre-blocked wet streptavidin agarose beads (Novagen). Pull-down was performed at 4°C for 16~20h with constant rotation, then sequentially washed with RIPA, Wash Buffer 1 (10mM Tris [pH 8.0], 200mM LiCl, 0.5% NP-40, 0.5% sodium deoxycholate, 1mM EDTA, 360mM NaCl), Wash Buffer 2 (Wash Buffer 1 without NaCl), and finally TE (10mM Tris pH = 7.6, 1mM EDTA). All washing was performed at 4°C for 10 min. with constant rotation. Each washing step was performed twice with the volume of the washing buffer at 10 times the volume of the dried beads. After the final wash, equal amount of 2X Laemmli sample buffer was added to the dried beads and boiled for 10min to elute the EdU-bound fraction for western blot analysis. For mass spectrometry, beads were eluted in 100mM Tris [pH 8.0], 1% SDS, 10mM DTT by boiling at 95° for 10 min.

### *In vivo* helicase assay

A schematic of this assay and examples of primary data are shown in S2 Fig. Cells were split and plated on two separate coverslips and cultured in the presence of 10μM BrdU. After 72h, the BrdU was removed. One of the coverslips ("EdU") was incubated in media containing 10μM EdU for 30min to label the replicating cells, while the other ("HU") was incubated in 3mM HU for 30min to induce replication fork stalling and allow the helicase to dissociate from the replisome and expose ssDNA in front of stalled forks. Then the EdU coverslip was stained for EdU (see section above on EdU incorporation section), while the HU coverslip was stained for BrdU under "native" conditions (no HCl denaturation of DNA, S2B Fig bottom panels). Positive controls for both parallel conditions were performed in which BrdU staining was performed following HCl denaturation of DNA, which demonstrates labeling of all cells due to the initial culturing in BrdU for 72 hours (S2B Fig top panels). Nuclei were counterstained with Hoechst 33342. Mounted coverslips were then examined by fluorescence microscopy. The percentage of BrdU-positive cells on the HU coverslip corresponds to the percentage of cells with sufficient helicase unwinding activity following fork arrest to expose ssDNA to a degree that allows detection (BrdU foci) over background. Comparison of this fraction to that of EdU-positive cells on the EdU coverslip reveals the percentage of replicating cells with active helicase unwinding activity.

### SV40 immortalization

An SV40 large T antigen-encoding construct (pBABE-puro SV40 LT) was packaged into lentivirus particles and used to infect primary MEFs at early passages. Cells were then selected and maintained in media containing 1.25μg/mL puromycin. Four pairs of littermate MEFs were transformed with this method and used for Dm-ChP.

### Small RNA sequencing and data analysis

Total RNA including small RNA was extracted using an miRNAeasy kit (Qiagen). One μg of total RNA from each sample was used to prepare small RNA sequencing libraries using the TruSeq small RNA sample preparation kit (index set 1–12, Illumina) according to the manufacturer’s instructions. Prepared libraries were sequenced on the Illumina HiSeq platform using single-end High Output mode. Reads were aligned to miRBase database v19 to generate read counts for each miRNA. Normalized miRNA reads for each sample were used as input for data analysis using the DESeq package [66]. Results are presented as supplementary information (S1 Dataset).

### *In silico* prediction of miRNA targets

To determine the potential miRNAs that target at *Mcm2-7* mRNAs, we used a combination of miRmap (http://mirmap.ezlab.org/) and miRanda (http://www.microrna.org/) software.

### Transfection of miRNA mimics

Primary WT MEFs were transfected with 50nM of miRNA mimic (Dharmacon) using DharmaFECT 1 transfection reagent per manufacturer's instructions. Control cells were transfected in parallel with negative control miRNA mimics (based on cel-miR-67), which has minimal sequence similarity with miRNAs in mice. 48h after transfection, cells were harvested for RNA and protein analysis.

### Luciferase assay

Schematic of luciferase assay is shown in S5A Fig. In general, HeLa cells were cotransfected using Lipofectamine 2000 in a 96 well format with 50nM of miRNA mimic and 100ng of pmirGLO luciferase construct with or without a 3’UTR insert. The 3’UTRs of *Mcm2-7* were cloned into *Nhe*I + *Sbf*I digested pmirGLO vector. Empty vector was used as control. 24h after transfection, cells were changed to fresh media. After 48h of transfection luciferase activities were measured using the Dual Luciferase Assay System (Promega) and Synergy 2 Multi-Mode Reader (BioTek) per manufacturer's instructions. *Renilla* luciferase activity was normalized to *Firefly* luciferase activity in each well.

### Generation of *Chaos3*/*miR-34abc*-TKO compound mutants

The *miR-34abc* knockout alleles were acquired from Dr. A. Nikitin [61]. They were crossed to the *Chaos3* mutant (C3Heb/FeJ background) for at least 5 generations. Male breeders from each generation were selected based on congenic status as evaluated by the DartMouse speed congenic service. A list of genotyping primers is presented in S1 Table.

## Supporting Information

S1 Fig Average *Mcm2-7* and *Pcna* mRNA levels as measured by qRT-PCR from total RNA isolated from WT primary MEFs at each passage. *Mcm2-7* showed increased reduction comparing to *Pcna* mRNA with increased passage of primary WT MEFs. Error bar = SEM.
(TIF)

S2 Fig *In vivo* helicase activity assay. **(A)** Flow chart of *in vivo* helicase activity assay. Refer to Methods section for a detailed explanation of the procedure. **(B)** Representative images of cultured cells subjected to the in vivo helicase activity assay. Top panels demonstrate successful BrdU incorporation into genomic DNA in all the cells during a 72h pulse-labeling period. The same cells do not stain for BrdU under non-denaturing conditions (middle panels). In the bottom panels, a high concentration of HU was added to stall replication forks. BrdU foci under native staining conditions revealed ssDNA between stalled replication forks and helicase that dissociated from the replisome and continued to unwind BrdU-containing genomic DNA.
(TIF)

S3 Fig Effects of chronic replication stress (HU-treatment) upon WT primary MEFs. **(A)** Proliferation of WT primary MEFs treated with HU. Relative cell number is the percentage vs. the untreated group on day1 (considered to be 100%). Error bar = SEM. **(B)** Persistent low level RS induces progressive loss of DNA replication in WT primary MEFs. The percentage of cells pulse-labeled with EdU (done immediately after HU removal) is presented. In the short term (24h), HU promotes EdU incorporation (**, p ≈ 5x10-5, two-sided t-test). However, long-term HU exposure (72h) eroded DNA replication potential significantly (*, p ≈ 0.001, two-sided t-test). N.S. = not significant. **(C)** Persistent RS induces MCM repression. *Mcm2-4* mRNA levels in WT primary MEFs were measured by qRT-PCR following 200μM HU treatment for the indicated periods of time. The values plotted are compared to untreated cells. Error bar = SEM.
(TIF)

S4 Fig Replicative lifespan of MCM2 gene-trap mutant (M2) and WT littermate primary MEFs. Cells were maintained under atmospheric O_2_ (~20%). Error bar = SEM.
(TIF)

S5 Fig Regulation of *Mcm2-7* by miRNAs. **(A)** Schematic of luciferase assay. Luciferase construct with 3’UTR of interest attached is subjected to miRNA control. If a miRNA targets the 3’UTR, it will repress luciferase protein production. The light signal strength ratio (no miRNA vs. miRNA) represents level of miRNA-mediated suppression. **(B)** Individual *miR-10b*, *27b* & *181a* overexpression through miRNA mimic transfection did not reduce *Mcm2-7* mRNA expression. mRNA levels were measured by qRT-PCR and normalized to β-actin levels. *Mcm2-7* mRNA levels were considered 100% in the control cells which were transfected with negative control miRNA mimics (based on *cel-miR-67*). Error bar = SEM. **(C)** *Mcm5*-siRNA knockdown caused reduced expression of other MCM mRNAs. 50nM siRNA specifically to *Mcm5* was transfected into primary WT MEFs and incubated for 48h. mRNA levels of *Mcm2-7* were measured by qRT-PCR and normalized to β-actin levels. *Mcm2-7* mRNA levels were considered to be 100% in the control cells which were mock transfected.
(TIF)

S6 Fig Micronucleus (MN) levels in mice bearing various *Chaos3* and *miR-34abc* genotypes. (TIF)

S1 Table PCR oligonucleotides used in study. (PDF)

S1 Dataset MicroRNA-seq data. (XLSX)
