# Supplementary figures and images for: Chronic DNA Replication Stress Reduces Replicative Lifespan of Cells by TRP53-Dependent, microRNA-Assisted MCM2-7 Downregulation
Source: PLoS Genet. 2016 Jan 14;12(1):e1005787. doi: 10.1371/journal.pgen.1005787 (PMC4713100; doi:10.1371/journal.pgen.1005787)

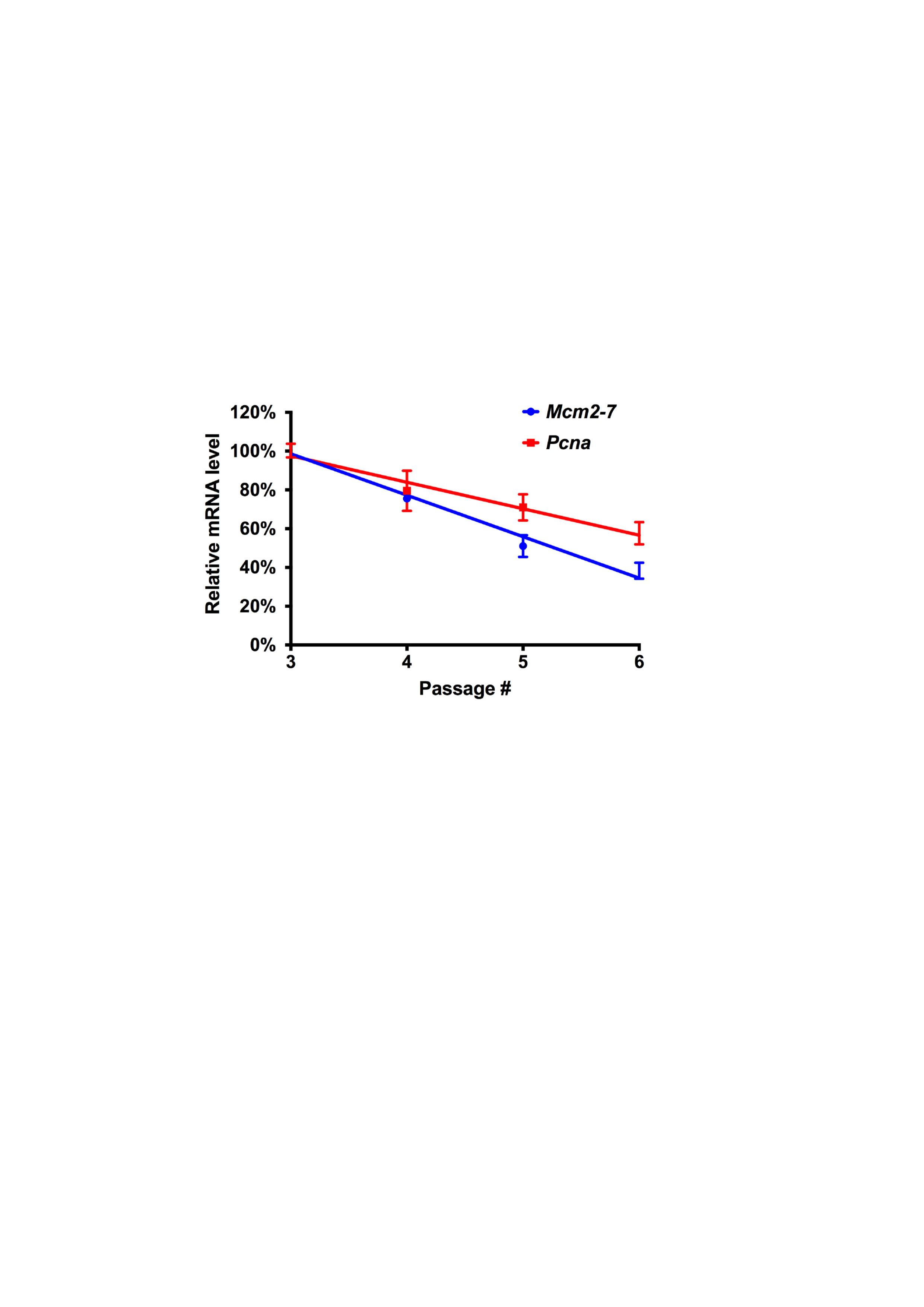

Supplement: S1 Fig — Mcm2-7 showed increased reduction comparing to Pcna mRNA with increased passage of primary WT MEFs. Error bar = SEM. (TIF) [file pgen.1005787.s001.tif]

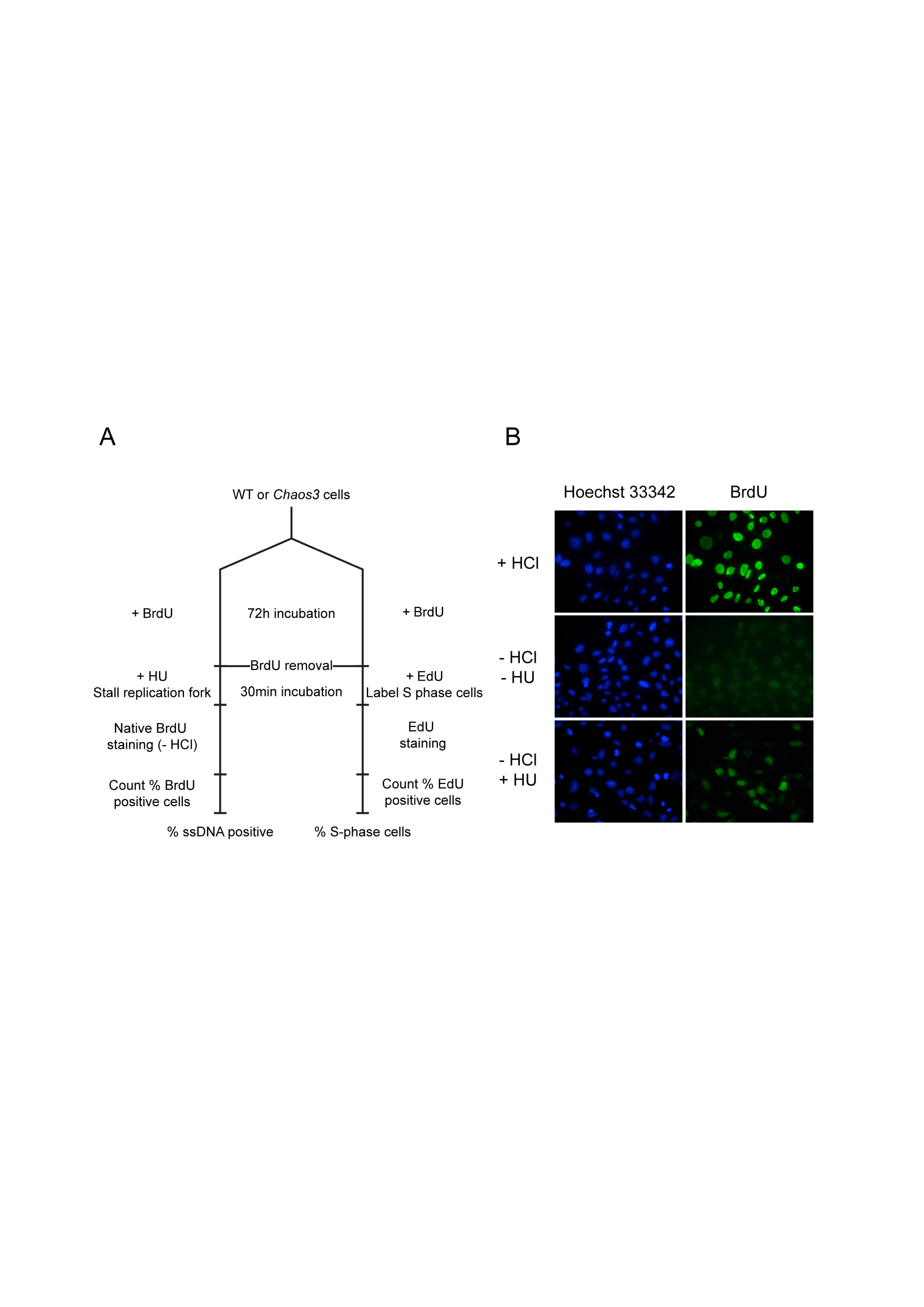

Supplement: S2 Fig — (A) Flow chart of in vivo helicase activity assay. Refer to Methods section for a detailed explanation of the procedure. (B) Representative images of cultured cells subjected to the in vivo helicase activity assay. Top panels demonstrate successful BrdU incorporation into genomic DNA in all the cells during a 72h pulse-labeling period. The same cells do not stain for BrdU under non-denaturing conditions (middle panels). In the bottom panels, a high concentration of HU was added to stall replication forks. BrdU foci under native staining conditions revealed ssDNA between stalled replication forks and helicase that dissociated from the replisome and continued to unwind BrdU-containing genomic DNA. (TIF) [file pgen.1005787.s002.tif]

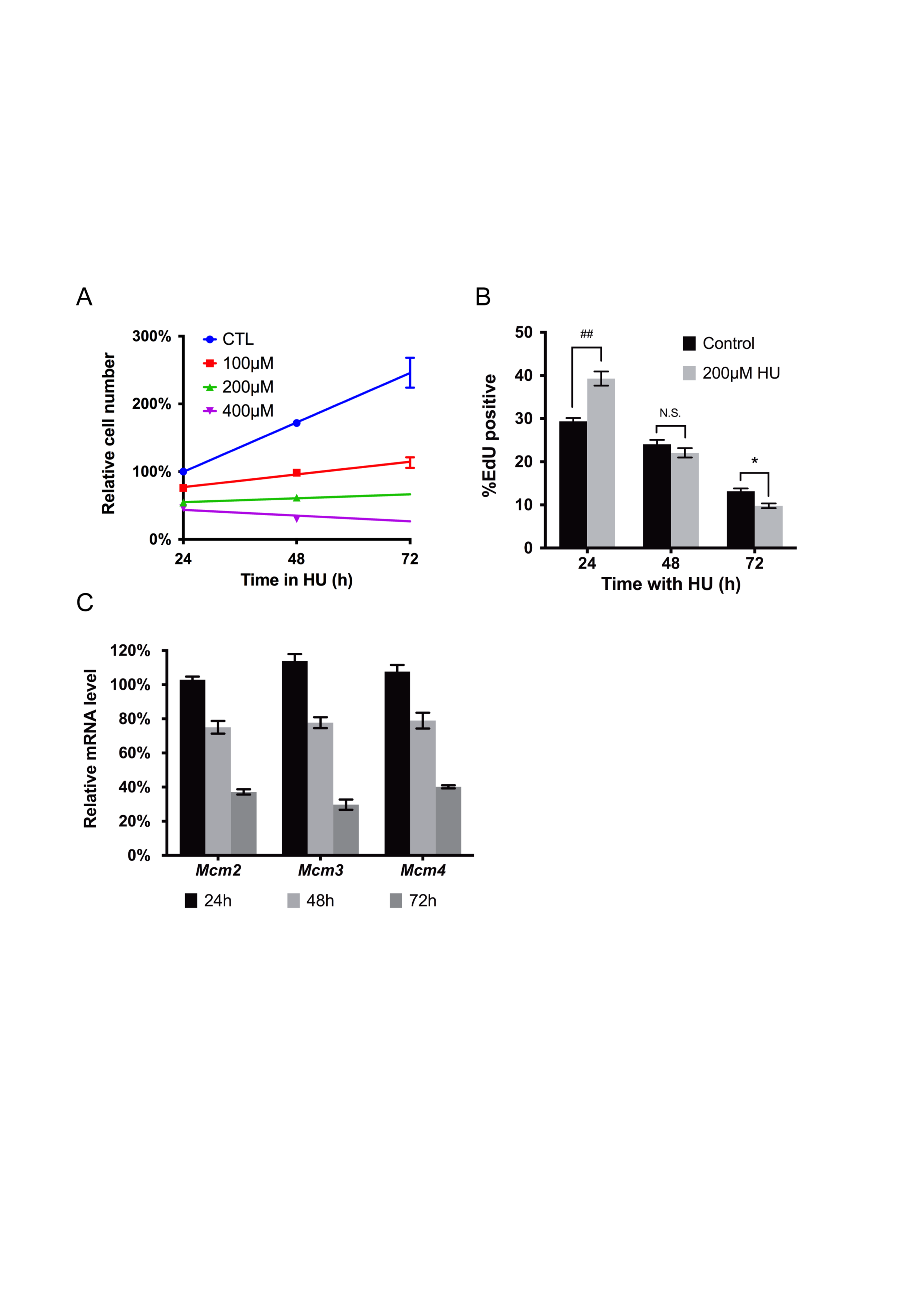

Supplement: S3 Fig — (A) Proliferation of WT primary MEFs treated with HU. Relative cell number is the percentage vs. the untreated group on day1 (considered to be 100%). Error bar = SEM. (B) Persistent low level RS induces progressive loss of DNA replication in WT primary MEFs. The percentage of cells pulse-labeled with EdU (done immediately after HU removal) is presented. In the short term (24h), HU promotes EdU incorporation (**, p ≈ 5x10^-5^, two-sided t-test). However, long-term HU exposure (72h) eroded DNA replication potential significantly (*, p ≈ 0.001, two-sided t-test). N.S. = not significant. (C) Persistent RS induces MCM repression. Mcm2-4 mRNA levels in WT primary MEFs were measured by qRT-PCR following 200μM HU treatment for the indicated periods of time. The values plotted are compared to untreated cells. Error bar = SEM. (TIF) [file pgen.1005787.s003.tif]

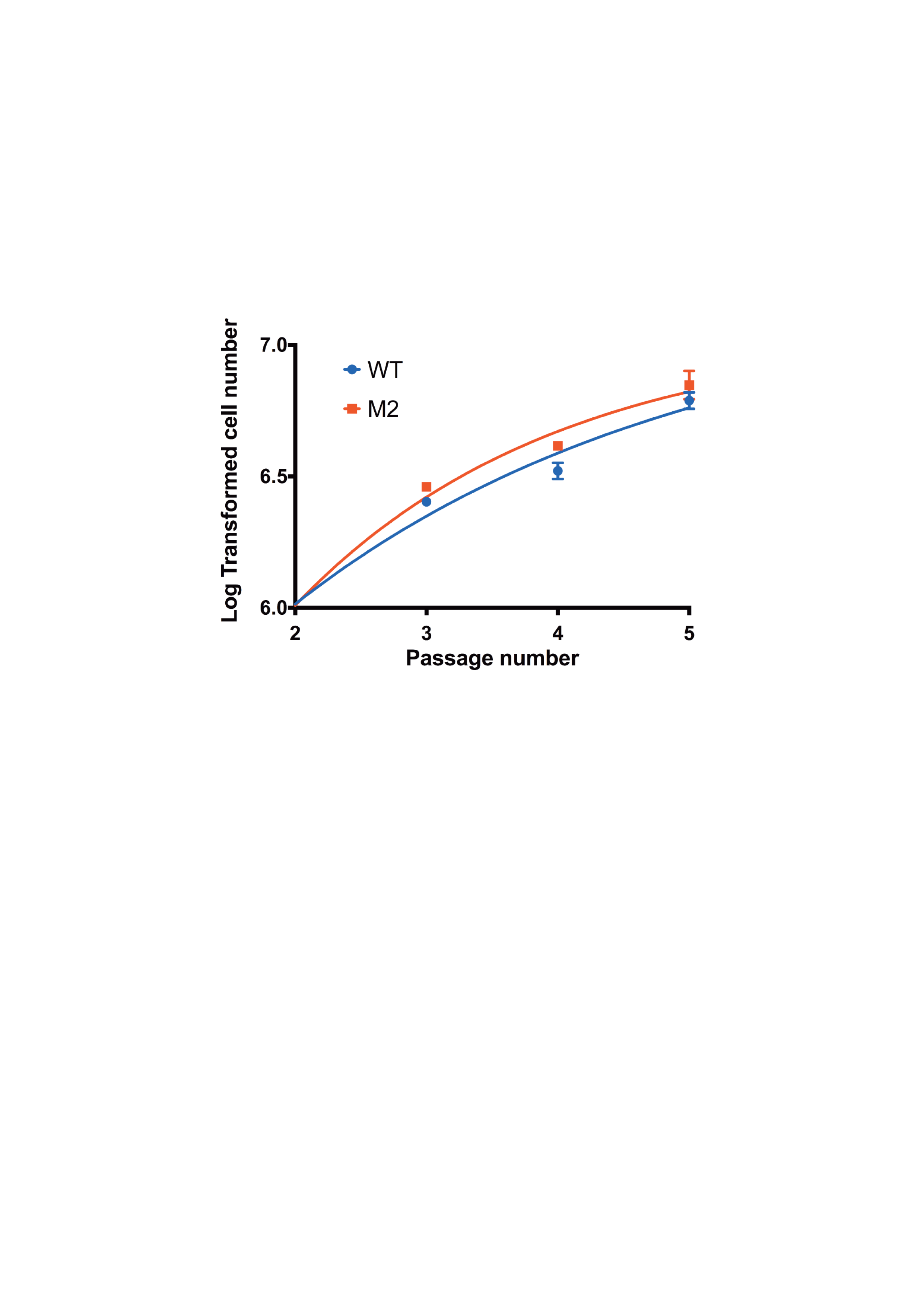

Supplement: S4 Fig — Cells were maintained under atmospheric O2 (~20%). Error bar = SEM. (TIF) [file pgen.1005787.s004.tif]

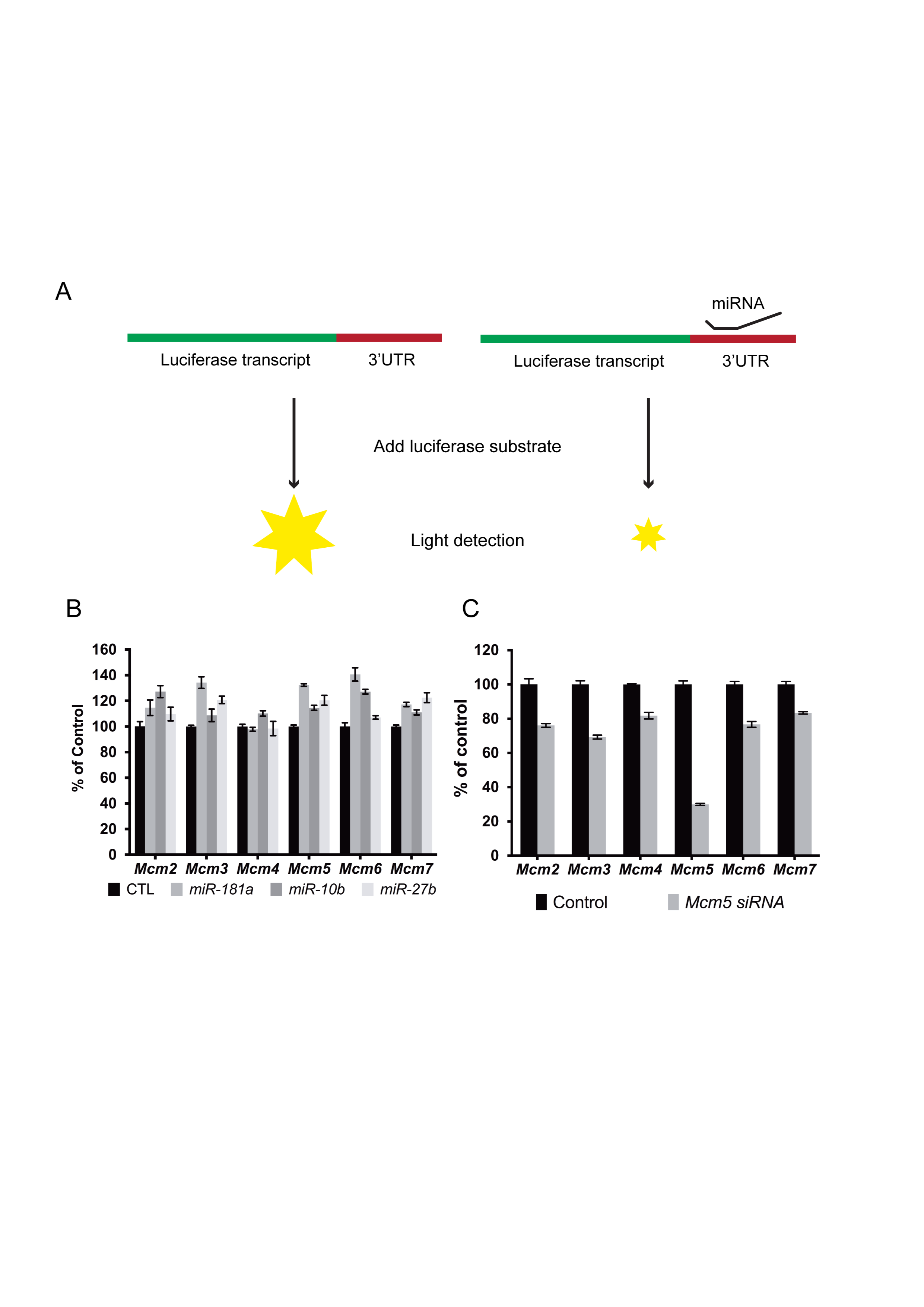

Supplement: S5 Fig — (A) Schematic of luciferase assay. Luciferase construct with 3’UTR of interest attached is subjected to miRNA control. If a miRNA targets the 3’UTR, it will repress luciferase protein production. The light signal strength ratio (no miRNA vs. miRNA) represents level of miRNA-mediated suppression. (B) Individual miR-10b, 27b & 181a overexpression through miRNA mimic transfection did not reduce Mcm2-7 mRNA expression. mRNA levels were measured by qRT-PCR and normalized to β-actin levels. Mcm2-7 mRNA levels were considered 100% in the control cells which were transfected with negative control miRNA mimics (based on cel-miR-67). Error bar = SEM. (C) Mcm5-siRNA knockdown caused reduced expression of other MCM mRNAs. 50nM siRNA specifically to Mcm5 was transfected into primary WT MEFs and incubated for 48h. mRNA levels of Mcm2-7 were measured by qRT-PCR and normalized to β-actin levels. Mcm2-7 mRNA levels were considered to be 100% in the control cells which were mock transfected. (TIF) [file pgen.1005787.s005.tif]

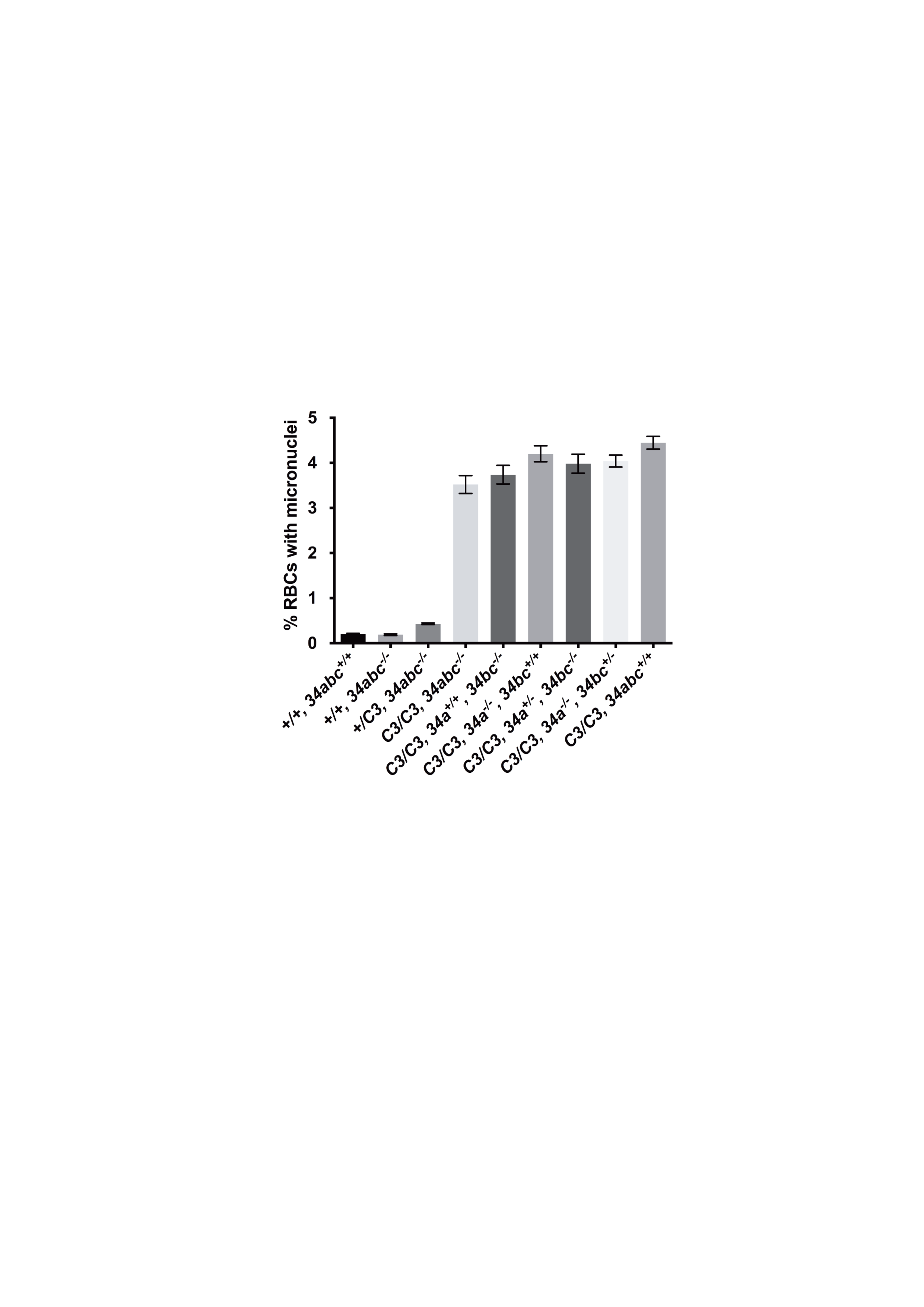

Supplement: S6 Fig — (TIF) [file pgen.1005787.s006.tif]
