# Supplementary material for: Chronic DNA Replication Stress Reduces Replicative Lifespan of Cells by TRP53-Dependent, microRNA-Assisted MCM2-7 Downregulation
Source: PLoS Genet. 2016 Jan 14;12(1):e1005787. doi: 10.1371/journal.pgen.1005787 (PMC4713100; doi:10.1371/journal.pgen.1005787)
Supplement: S1 Table — (PDF) [file pgen.1005787.s007.pdf]

# Supplementary Tabel

| Name | Sequence (5' to 3') | Purpose |
|---|---|---|
| Chaos3typeL | CATTGATCAGCTCATCACCA | Chaos3 mutant genotyping |
| Chaos3typeR | CACATACCATTTGCTTGTCAG | |
| Mcm2-GT-F | CCCTCCTCCTGCAGGTGGAAAGCAC | Mcm2 gene-trap genotyping |
| Mcm2-GT-R1 | GCAGTAGAGTTCCCAGGAGGAGCC | |
| Mcm2-GT-R2 | GGTGGTGTAAGGAACAGATGGAC | |
| Mcm2 mRNA-F | TTCCCGCTTTGATGTCCTG | Mcm2 qRT-PCR |
| Mcm2 mRNA-R | ACCATTAGTCAACCCTTCATCC | |
| Mcm3 mRNA-F | AGGAAGACTCATGCCAAGGATGGA | Mcm3 qRT-PCR |
| Mcm3 mRNA-R | TGGGCTCACTGAGTTCCACTTTCT | |
| Mcm4 mRNA-F | ACAGGAATGAGTGCCACTTCTCGT | Mcm4 qRT-PCR |
| Mcm4 mRNA-R | AAAGCTCGCAGGGCTTCTTCAAAC | |
| Mcm5 mRNA-F | CTGGATGCTGCTTTGTCTGGCAAT | Mcm5 qRT-PCR |
| Mcm5 mRNA-R | TGTGTTCAGACACCTGAGAGCCAA | |
| Mcm6 mRNA-F | TCACCAAGTCCTCGTGGAGAATCA | Mcm6 qRT-PCR |
| Mcm6 mRNA-R | TTTAGGCTGAACCTCGTCACAGCA | |
| Mcm7 mRNA-F | CCCTGCCCAATTTGAACCTTTGGA | Mcm7 qRT-PCR |
| Mcm7 mRNA-R | TCTCCACATATGCTGCGGTGATGT | |
| Pcna mRNA-F | GGGTTGGTAGTTGTCGCTGT | Pcna qRT-PCR |
| Pcna mRNA-R | TCCAGCACCTTCTTCAGGAT | |
| Cdc6 mRNA-F | GCTGCCCTGGACTTTTTAAG | Cdc6 qRT-PCR |
| Cdc6 mRNA-R | GCTGCTTGACTCGGATATGA | |
| Cdkn2a-qF | ACATCAAGACATCGTGCGATATT | Cdkn2a qRT-PCR |
| Cdkn2a-qR | CGGTACACAAAGACCACCC | |
| mp16INK4a-qF | AATCTCCGCGAGGAAAGCGAACT | p16-Ink4a qRT-PCR |
| mp16INK4a-qR | GTGAACGTTGCCCATCATCATCACC | |
| mp19ARF-qF | CGTGAACATGTTGTTGAGGCTAGAGA | p19-ARF qRT-PCR |
| mp19ARF-qR | TCTGCACCGTAGTTGAGCAGAAGAG | |
| ActB mRNA-F | ACCTTCTACAATGAGCTGCG | beta-Actin qRT-PCR |
| ActB mRNA-R | CTGGATGGCTACGTACATGG | |
| Cdk2 mRNA-F | ATGAGGTGGTTTGGCCAGGAGTTA | Cdk2 qRT-PCR |
| Cdk2 mRNA-R | CTGCTTTGGCTGAAATCCGCTTGT | |
| Ccne1 mRNA-F | CCTCCAAAGTTGCACCAGTTTGCT | Cyclin E1 qRT-PCR |
| Ccne1 mRNA-R | TCGTTGACATAGGCCACTTGGACA | |
| Ccne2 mRNA-F | GACGCAGTAGCCGTTTACAA | Cyclin E2 qRT-PCR |
| Ccne2 mRNA-R | ATAATGCAAGGGCTGATTCC | |
| Ccna2 mRNA-F | CTTGGCTGCACCAACAGTAA | Cyclin A2 qRT-PCR |
| Ccna2 mRNA-R | ATGACTCAGGCCAGCTCTGT | |

| Name | Sequence (5' to 3') | Purpose |
|---|---|---|
| miR-34a_A | GAATGTGTATACGTGTTTTGCCTGA | miR-34a genotyping |
| miR-34a_F | TGGCCCCTTTAATTTACAAGCCCA | |
| miR-34a_D | AGCTGACATGCCAGGAATGCTGA | |
| miR-34bc_A | CTGCGCTTCTTTCTTCGATGTAGC | miR-34bc genotyping |
| miR-34bc_B | TGGCTTTAGGATCTCCATTTCAGC | |
| miR-34bc_D | ACCTGGTTAAGTGGGCTGAGTTCC | |
| pmirGLO.Seq_F | GACGAGGTGCCTAAAGGAC | Sequencing primers for pmirGLO |
| pmirGLO.Seq_R | CCAACTCAGCTTCCTTTCG | |
| Mcm2 3'UTR F | AAATTTGCTAGCGACCAGCACAGGGGCCTC | Primers for cloning full-length Mcm2 3'UTR into pmirGLO |
| Mcm2 3'UTR R | AAATTTCCTGCAGGTAGCGCTATTTAGGTTTTATTC | |
| Mcm3 3'UTR F | AAATTTGCTAGCAGTTGTTGCTACCAAGTAC | Primers for cloning full-length Mcm3 3'UTR into pmirGLO |
| Mcm3 3'UTR R | AAATTTCCTGCAGGTACAAAATAGTTATATTTACTCAGTATG | |
| Mcm4 3'UTR F | AAATTTGCTAGCGCTGCATGGCCCTCGGAC | Primers for cloning full-length Mcm4 3'UTR into pmirGLO |
| Mcm4 3'UTR R | AAATTTCCTGCAGGAATTCAAATGGTTTAGAGATTTATTG | |
| Mcm5 3'UTR F | AAATTTGCTAGCGCCCATTGCCCATCAACC | Primers for cloning full-length Mcm5 3'UTR into pmirGLO |
| Mcm5 3'UTR R | AAATTTCCTGCAGGGGTGGCCCACACTTTTAATCC | |
| Mcm6 3'UTR F | AAATTTGCTAGCGGTATTGAAGGTAACTTGATGG | Primers for cloning full-length Mcm6 3'UTR into pmirGLO |
| Mcm6 3'UTR R | AAATTTCCTGCAGGCACAGAACAAGTTGTTTTATTTTCATG | |
| Mcm7 3'UTR F | AAATTTGCTAGCTAGCCAGTTTTTACACCCTCC | Primers for cloning full-length Mcm7 3'UTR into pmirGLO |
| Mcm7 3'UTR R | AAATTTCCTGCAGGAACAAGCAAGAGGCAATCAAAAC | |
